# LC3/GABARAPs drive ubiquitin-independent recruitment of Optineurin and NDP52 to amplify mitophagy

**DOI:** 10.1038/s41467-019-08335-6

**Published:** 2019-01-24

**Authors:** Benjamin Scott Padman, Thanh Ngoc Nguyen, Louise Uoselis, Marvin Skulsuppaisarn, Lan K. Nguyen, Michael Lazarou

**Affiliations:** Department of Biochemistry and Molecular Biology, Biomedicine Discovery Institute, Monash University, Melbourne, 3800 Australia

## Abstract

Current models of selective autophagy dictate that autophagy receptors, including Optineurin and NDP52, link cargo to autophagosomal membranes. This is thought to occur via autophagy receptor binding to Atg8 homologs (LC3/GABARAPs) through an LC3 interacting region (LIR). The LIR motif within autophagy receptors is therefore widely recognised as being essential for selective sequestration of cargo. Here we show that the LIR motif within OPTN and NDP52 is dispensable for Atg8 recruitment and selectivity during PINK1/Parkin mitophagy. Instead, Atg8s play a critical role in mediating ubiquitin-independent recruitment of OPTN and NDP52 to growing phagophore membranes via the LIR motif. The additional recruitment of OPTN and NDP52 amplifies mitophagy through an Atg8-dependent positive feedback loop. Rather than functioning in selectivity, our discovery of a role for the LIR motif in mitophagy amplification points toward a general mechanism by which Atg8s can recruit autophagy factors to drive autophagosome growth and amplify selective autophagy.

## Abstract (web summary)

Selective autophagy receptors are thought to selectively recruit Atg8 positive membranes to cargo via their LIR motif. Here, the authors show the LIR motifs in OPTN and NDP52 are dispensable for selectivity, functioning instead to recruit additional receptors and amplify mitophagy.

## Introduction

The Parkinson’s disease proteins, PINK1 and Parkin, mediate ubiquitin-dependent clearance of damaged mitochondria through a selective form of autophagy termed mitophagy^1–4^. During mitophagy, PINK1 phosphorylates both ubiquitin and Parkin at S65 to recruit and activate Parkin’s ubiquitin ligase activity on damaged mitochondria^5–7^. Once active, Parkin conjugates ubiquitin chains onto mitochondrial outer membrane proteins. PINK1 phosphorylates the ubiquitin chains at S65 (pS65-Ub) to promote further rounds of Parkin recruitment and activation in a positive feedback loop^8–13^. This results in rapid coating of mitochondria with pS65-Ub chains that function to link damaged mitochondria to the autophagy machinery by recruiting the primary mitophagy receptors OPTN and NDP52^14–17^.

OPTN and NDP52 belong to a group of ubiquitin binding autophagy receptors which includes p62, NBR1 and TAX1BP1. After binding to ubiquitinated cargo through ubiquitin binding domains (UBD), the autophagy receptors are widely thought to then link cargo to autophagosomal membranes via binding to Atg8 family proteins. In humans, the Atg8 family consists of six primary members belonging to the LC3 (LC3A, LC3B and LC3C) and GABARAP subfamilies (GABARAP, GABARAPL1 and GABARAPL2). Autophagy receptors bind to Atg8 proteins via a short peptide sequence known as the LC3-interacting region (LIR), which binds to a pair of conserved hydrophobic pockets common to all mammalian Atg8 homologs^18,19^. The LIR motif within autophagy receptors is a central feature of canonical selective autophagy models, in which the autophagy receptors mediate recruitment of Atg8-positive membranes^18,20,21^. The Atg8-positive membranes are then thought to expand around targeted cargoes thereby selectively encapsulating them within autophagosomes^20–24^. In addition to Atg8 binding, OPTN and NDP52 can also function by promoting the recruitment of the unc51-like activating kinase 1/2 complex (ULK1/2-Atg13-FIP200-Atg101) during PINK1/Parkin mitophagy^14^. The ULK1 complex initiates autophagosome formation on the surface of damaged mitochondria and drives the downstream activation of the phosphoinositide 3-kinase (PI3K) complex^25–28^. The PI3K complex generates phosphatidylinositol 3-phosphate (PtdIns(3)P)^29^, which recruits effector proteins including WIPI1/2, and the E3-like conjugation machinery (Atg12-Atg5-Atg16L1 complex) that conjugates Atg8s onto autophagosomal membranes^29–34^.

Atg8 family members are required for efficient autophagosome formation and can regulate autophagosome size^35–38^. Despite their importance during PINK1/Parkin mitophagy, recent evidence indicates that the Atg8 family does not play an essential role in selective sequestration of mitochondria. Cells lacking Atg8s can successfully sequester mitochondria within autophagosomes^38^, and Atg8 lipidation deficient cells also contain autophagic membranes surrounding mitochondria^30^. Given that Atg8s do not mediate selective recognition, the role of LIR-mediated interactions between autophagy receptors and Atg8 family members during PINK1/Parkin mitophagy is unclear.

In this study, we discover that the LIR motif within OPTN or NDP52 is not essential for either LC3 or GABARAP subfamily recruitment. However, the LIR motif plays an important role in driving ubiquitin-independent recruitment of OPTN and NDP52 to autophagic membranes following autophagosome initiation. The post-initiation recruitment of OPTN and NDP52 promotes additional ULK1 complex recruitment resulting in an amplification of autophagosome biogenesis and mitophagy. All five of the major ubiquitin binding autophagy receptors are capable of LIR-mediated recruitment via Atg8s, but only OPTN and NDP52 robustly amplify PINK1/Parkin mitophagy. In silico modelling supports the existence of an Atg8-dependent positive feedback loop of autophagy receptor recruitment and autophagosome formation that amplifies mitophagy. This may represent a general mode of signal amplification in other selective autophagy pathways.

## Results

### Analysis of the LIR motif during PINK1/Parkin mitophagy

To determine the role of the LIR motif during PINK1/Parkin mitophagy, we first assessed the Atg8 recruitment profile of OPTN and NDP52 following PINK1/Parkin activation. LIR amino acid sequences vary between autophagy receptors, with different Atg8 binding specificities reported for each protein. For example, the LIR motif in OPTN has a high affinity for GABARAP^39^, but the affinity can switch toward LC3B when OPTN is phosphorylated at S177 by TANK binding kinase (TBK1)^40^. NDP52 contains a non-canonical LIR motif (also termed CLIR) that is highly selective for LC3C binding^23^. To assess the Atg8 recruitment profile of OPTN and NDP52 during PINK1/Parkin mitophagy, we utilised penta knockout (KO) cells which lack the five major autophagy receptors (OPTN, NDP52, TAX1BP1, NBR1 and p62)^14^.

**Fig. 1:**
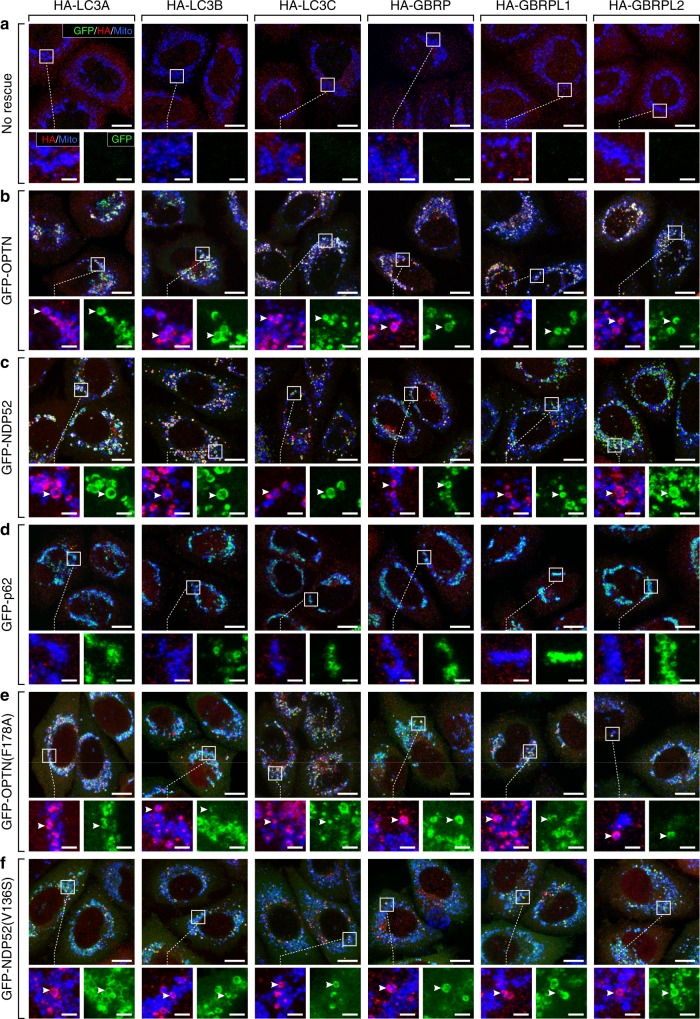
The LIR motif within autophagy receptors is dispensable for Atg8 recruitment during PINK1/Parkin mitophagy. **a**–**f** Representative immunofluorescence images of penta KO HeLa cells stably expressing untagged Parkin and either HA-LC3A, HA-LC3B, HA-LC3C, HA-GBRP, HA-GBRPL1 or HA-GBRPL2, without receptor expression (**a**), or rescued by co-expression of either GFP-OPTN (**b**), GFP-NDP52 (**c**), GFP-p62 (**d**), GFP-OPTN(F178A) (**e**) or GFP-NDP52(V136S) (**f**); immunostained for HSP60, HA & GFP after 3 h OA treatment. Atg8-positive mitochondria are indicated by arrowheads. Scale bars: overviews, 10 µm; insets, 2 µm

Atg8 localization was assessed using HA-tagged variants of all six mammalian Atg8s (LC3A, LC3B, LC3C, GBRP, GBRPL1 and GBRPL2), to avoid the functional impairment associated with GFP-tagged LC3/GABARAPs^14,38^. Penta KO cell lines stably expressing untagged Parkin and individual HA-Atg8s were rescued with either GFP-OPTN or GFP-NDP52, before treatment with oligomycin and antimycin A (OA) for 3 h to activate PINK1/Parkin mitophagy. HA-tagged Atg8s were not recruited to mitochondria in the absence of autophagy receptor expression (Fig. 1a), whereas all HA-tagged Atg8s were recruited in penta KOs rescued with either GFP-OPTN or GFP-NDP52 (Fig. 1b, c). Neither OPTN nor NDP52 displayed a preference for a particular Atg8 family member during mitophagy (Fig. 1b, c).

To confirm that Atg8 recruitment was dependent on the LIR motif, we analysed penta KO cells rescued with LIR motif mutant GFP-OPTN(F178A), or GFP-NDP52(V136S). Unexpectedly, both GFP-OPTN(F178A) and GFP-NDP52(V136S) could still recruit all HA-tagged Atg8s (Fig. 1e, f). Co-immunoprecipitation experiments confirmed that the LIR point mutants of OPTN and NDP52 prevented autophagy receptor interaction with Atg8s (Supplementary Fig. 1a, b). OPTN(F178A) binding to HA-LC3B (Supplementary Fig. 1a) and NDP52(V136S) binding to HA-LC3C (Supplementary Fig. 1b) were inhibited. Despite their inability to bind Atg8 family members, LIR-mutant OPTN(F178A) and NDP52 (V136S) both significantly recruited HA-LC3B and HA-LC3C, respectively, following PINK1/Parkin mitophagy activation (Supplementary Fig. 1c, d). In contrast, penta KO cells rescued with GFP-p62 failed to recruit any Atg8s despite the presence of a functional LIR motif within p62 (Fig. 1d). The LIR motif within autophagy receptors is therefore dispensable for both Atg8 recruitment and selective recognition of mitochondria. The ULK1 autophagy initiation complex can be recruited by NDP52 and OPTN, but not p62^14^. Thus, the recruitment of the ULK1 complex and activation of downstream autophagy machineries is likely to govern Atg8 recruitment^32,34^.

### The LIR motif promotes efficient mitophagy

**Fig. 2:**
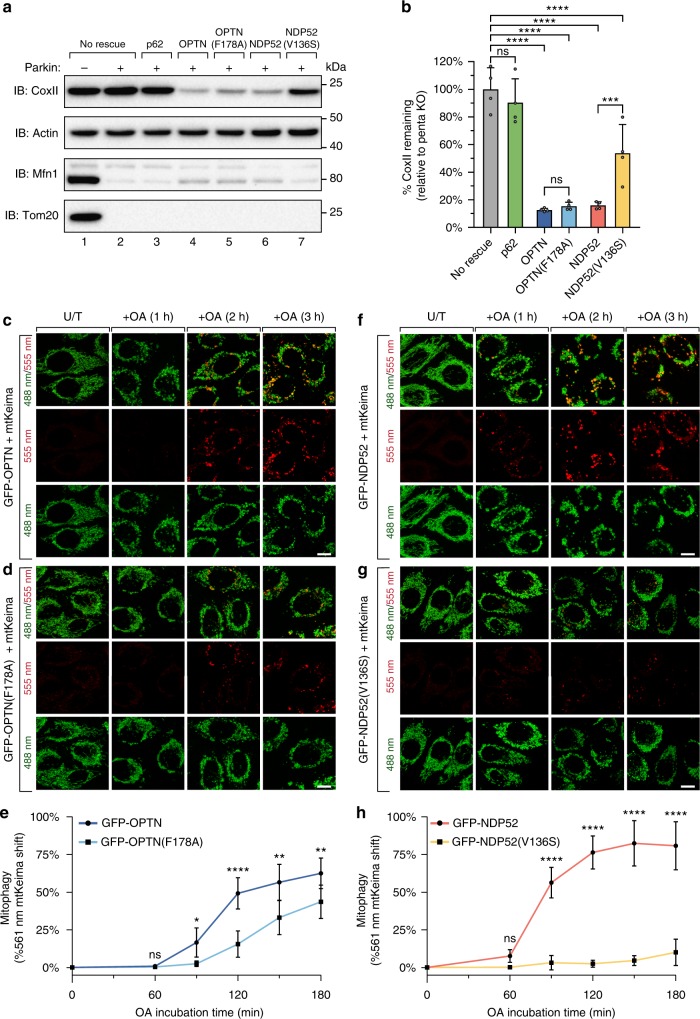
The LIR motif within OPTN and NDP52 is essential for efficient mitophagy. **a**, **b** Penta KO HeLa cells with or without untagged Parkin expression rescued by stable expression of the indicated GFP-tagged receptors were analysed by immunoblotting after 21 h incubation with OA (**a**) for quantification of the remaining CoxII levels (**b**). **c**–**h**, penta KO HeLa cells stably expressing mtKeima, untagged Parkin and either GFP-OPTN (**c**), GFP-OPTN(F178A) (**d**), GFP-NDP52 (**f**) or GFP-NDP52(V136S) (**g**), were imaged by live-cell confocal microscopy ((**c**, **d**, **f**, **g**); GFP-receptor channels provided in Supplementary Fig. 1g, h), and analysed by Fluorescence Activated Cell Sorting (FACS) to quantify the percentage of 561 nm mtKeima-positive cells (**e**, **h**), after time-course incubation with OA. (Representative FACS plots provided in Supplementary Fig. 1j; FACS analysis and representative images for penta KO & GFP-p62 provided in Supplementary Fig. 1e, f, i). Data in (**b**) are mean ± s.d. from four independent experiments. Data in (**e**, **h**) are mean ± s.d. from three independent experiments. *P < 0.05, **P < 0.005, ***P < 0.001, ****P < 0.0001 (**b** one-way ANOVA; **e**, **h** two-way ANOVA). ns: not significant. Scale bars: 10 µm

Our results reveal that the LIR motif is not essential for Atg8 family recruitment nor selectivity, however, several studies have demonstrated the importance of the LIR motif for selective autophagy^17,23,24^. We therefore assessed the mitophagy activity of LIR-mutant GFP-OPTN(F178A) and GFP-NDP52(V136S). Penta KO cells rescued with GFP-tagged WT and LIR-mutant autophagy receptors, or p62 as a negative control^14^, were treated with OA for 21 h before immunoblotting for the mitochondrial DNA encoded protein CoxII to assess mitophagy (Fig. 2a, b). LIR-mutant GFP-OPTN(F178A) robustly degraded CoxII similarly to WT GFP-OPTN, whereas GFP-NDP52(V136S) had a significant mitophagy defect relative to WT GFP-NDP52. Despite the mitophagy deficiency observed for LIR-mutant GFP-NDP52(V136S), it could still drive mitophagy to a significant degree when compared to the penta KO control which lacks autophagy receptor expression (Fig. 2a; compare lanes 2 and 7, 2b). The ability of OPTN(F178A) and NDP52(V136S) to drive CoxII degradation indicates that the LIR motif is not essential for mitochondrial clearance. However, the decrease in mitophagy levels observed for NDP52(V136S) pointed toward a defect in mitophagy efficiency. Given that mitophagy efficiency defects may only be apparent at earlier time points^24^, we utilised mtKeima to measure mitochondrial delivery to lysosomes during the first 3 h of OA incubation (Fig. 2c–h; Supplementary Fig. 1j). Indeed, LIR-mutant GFP-OPTN(F178A) was observed to have a significant defect in the rate of mitophagy (Fig. 2c–e), while GFP-NDP52(V136S) had a much greater mitophagy defect (Fig. 2f–h) consistent with the CoxII degradation data (Fig. 2a, b). Expression of GFP-p62 failed to rescue mitophagy in any of the conducted assays (Fig. 2b; Supplementary Fig. 1f, i). Thus, the LIR motif within OPTN and NDP52 functions to drive efficient mitochondrial clearance during PINK1/Parkin mitophagy.

**Fig. 3:**
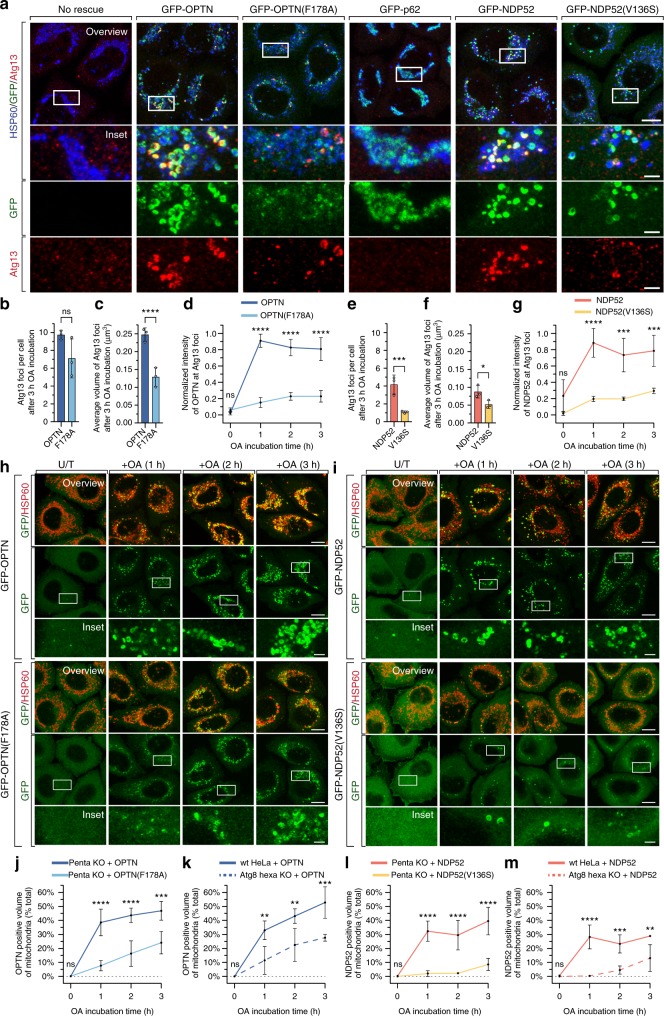
OPTN and NDP52 recruitment is influenced by LIR-mediated interactions with Atg8s. **a** Representative immunofluorescence images of penta KO HeLa cells stably expressing untagged Parkin rescued by expression of the indicated GFP-tagged receptors; immunostained for Atg13, HSP60 and GFP after treatment with OA for 3 h. (Equivalent images for TAX1BP1 & NBR1 are provided in Supplementary Fig. 5b). **b**–**g** Automated image analysis of the average Atg13 foci count per cell (**b, e**), Atg13 foci volume (**c**, **f**) and intensity of foci in the GFP channel (**d, g**), in penta KO HeLa cells stably expressing untagged Parkin, rescued by stable expression of either GFP-OPTN/GFP-OPTN(F178A) (**b**–**d**) or GFP-NDP52/GFP-NDP52(V136S) (**e**–**g**), immunostained for Atg13, HSP60 and GFP after time-course incubation with OA. (Data from other time points shown in Supplementary Fig. 2g-j). **h**, **i**, **j**, **l** Representative immunofluorescence images (**h**, **i**) and automated image analysis of GFP-tagged receptor translocation (**j**, **l**) in penta KO HeLa cells stably expressing untagged Parkin after rescue expression of either GFP-OPTN/GFP-OPTN(F178A) (**h, j**) or GFP-NDP52/GFP-NDP52(V136S) (**i**, **l**), immunostained for HSP60 and GFP after time-course incubation with OA (times indicated). **k**, **m** Automated image analysis of GFP-OPTN (**k**) or GFP-NDP52 (**m**) translocation in WT and hexa KO HeLa cells stably expressing untagged Parkin, after time-course incubation with OA and immunostaining for HSP60 & GFP. (Representative images for (**k**, **m**) provided in Supplementary Fig. 2q, r). Data in (**b**–**g**) and (**j**–**m**) are mean ± s.d. from three independent experiments. *P < 0.05, **P < 0.005, ***P < 0.001, ****P < 0.0001 (two-way ANOVA). ns: not significant. Scale bars: overviews, 10 µm; insets, 2 µm

To determine how the LIR motif might contribute to mitophagy efficiency, we analysed the stages of mitophagy following autophagy receptor recruitment. OPTN and NDP52 play a key role during autophagosome initiation by promoting the recruitment of the ULK1 complex^14^. We therefore quantified the formation of autophagosome initiation foci containing the ULK1 complex subunit Atg13^29^. Penta KO cells expressing WT or LIR-mutant autophagy receptors were immunolabelled for endogenous Atg13 after an OA treatment time-course. The analysis revealed that Atg13 foci in penta KO cells expressing GFP-OPTN(F178A) were significantly smaller than those in cells expressing WT GFP-OPTN (Fig. 3a–c), although the overall number of Atg13 foci was not significantly different (Fig. 3b). In contrast, cells expressing GFP-NDP52(V136S) contained both fewer and smaller Atg13 foci relative to cells expressing WT GFP-NDP52 (Fig. 3a, e, f). Recruitment of the initiation machinery to mitochondria by WT and LIR-mutant autophagy receptors was also analysed using subcellular fractionation (Supplementary Fig. 3f,g and i,j). Mitochondrial fractions from penta KO cells expressing OPTN(F178A) or NDP52(V136S) had reduced levels of the ULK1 complex subunit FIP200 relative to WT controls, corroborating the microscopy analyses. Consistent with the defects in Atg13 foci formation (Fig. 3a) and FIP200 recruitment (Supplementary Figure 3f–g and i–j), the recruitment of downstream autophagy effectors including WIPI2b (Supplementary Fig. 2a–c) and Atg16L1 (Supplementary Fig. 2d–f) were also defective in cells expressing LIR-mutant autophagy receptors. Penta KO cells either lacking autophagy receptor expression or expressing GFP-p62 failed to initiate mitophagy as demonstrated by a lack of Atg13 foci (Fig. 3a; Supplementary Fig. 2a, d). Importantly, our analyses of Atg13 foci also revealed that the intensity of GFP-OPTN(F178A) and GFP-NDP52(V136S) at phagophores was significantly reduced relative to WT GFP-OPTN (Fig. 3d) and GFP-NDP52 (Fig. 3g). Taken together, our results reveal that mutation of the LIR motif in OPTN and NDP52 causes an autophagosome formation defect at early stages of mitophagy including phagophore formation. In addition, the reduction of autophagy receptor intensity at phagophore formation sites indicates that mutation of the LIR motif affects events upstream and/or during autophagosome initiation.

We next asked whether the reduced intensity of LIR-mutant OPTN and NDP52 at autophagosome formation sites was caused by reduced autophagy receptor translocation. GFP-OPTN(F178A) (Fig. 3h, j) and NDP52(V136S) (Fig. 3i, l) translocated significantly slower than their wild-type counterparts. Mitochondrial translocation of NDP52(V136S) was particularly low, consistent with the greater mitophagy defect observed in penta KO cells expressing this mutant (Fig. 2). Defects in mitochondrial translocation of OPTN(F178A) and NDP52(V136S) were also detected via analysis of isolated mitochondrial fractions (Supplementary Fig. 3f–k). To confirm that reduced translocation of LIR-mutant OPTN and NDP52 was due to their inability to bind Atg8s, and to exclude the potential effect of protein alteration from mutated residues, we then assessed WT GFP-OPTN and GFP-NDP52 translocation rates in Atg8 hexa KO cells that lack all six LC3/GABARAPs (Fig. 3k, m; Supplementary Fig. 2q, r). The absence of Atg8 proteins severely impaired the translocation of both WT GFP-OPTN (Fig. 3k; Supplementary Fig. 2q) and GFP-NDP52 (Fig. 3m; Supplementary Fig. 2r), which displayed very similar translocation rates to the LIR mutants expressed in penta KO cells (Fig. 3j, l). Autophagy receptor translocation during PINK1/Parkin mitophagy is thought to be entirely dependent on binding to ubiquitin chains, with both OPTN and NDP52 previously shown to preferentially bind to ubiquitin chains phosphorylated at S65 by PINK1^14,17^. However, analysis of pS65-Ub levels on mitochondria in WT and hexa KO cells confirmed that hexa KO cells did not have reduced levels of pS65-Ub chains (Supplementary Fig. 3a), thus eliminating the possibility that altered pS65-Ub levels had affected autophagy receptor translocation. Collectively, these results demonstrate that OPTN and NDP52 recruitment efficiency during PINK1/Parkin mitophagy is directly influenced by LIR-motif-mediated interactions with LC3s and/or GABARAPs.

### LIR-mediated recruitment of autophagy receptors in mitophagy

**Fig. 4:**
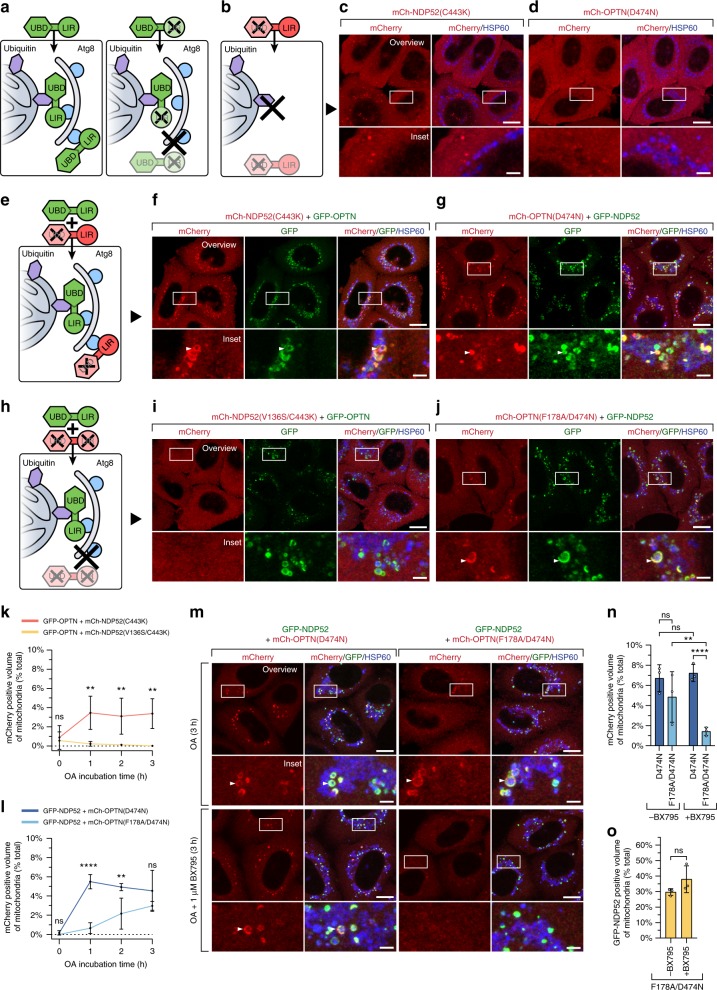
The LIR motif mediates autophagy receptor recruitment after initiation of autophagosome biogenesis. **a** Schematic of the proposed mechanism by which Atg8s influence receptor translocation, depicting wild-type receptor recruitment via ubiquitin and Atg8 proteins and LIR-mutant receptor recruitment exclusively via ubiquitin (the upstream stages of this model are in depicted in Supplementary Fig. 4a). **b**, **e**, **h** Model schematics depicting the predicted translocation outcomes in cells expressing a UBD mutant receptor alone (**b**), co-expressing a UBD mutant receptor and a wild-type receptor (**e**), or co-expressing a UBD/LIR double mutant receptor and a wild-type receptor (**h**). **c**, **d**, **f**, **g**, **i**, **j** Representative images of penta KO HeLa cells stably expressing untagged Parkin, with co-expression of either mCh-NDP52(C443K) (**c**), mCh-OPTN(D474N) (**d**), GFP-OPTN & mCh-NDP52(C443K) (**f**), GFP-NDP52 & mCh-OPTN(D474N) (**g**), GFP-OPTN and mCh-NDP52(V136S/C443K) (**i**), or GFP-NDP52 and mCh-OPTN(F178A/D474N) (**j**), after 3 h OA incubation and immunostaining for HSP60, GFP and mCherry. **k**, **l** Automated image analysis of mCherry translocation in the cell lines shown in (**f** and **i**) (**k**) or in (**g** and **j**) (**l**), after time-course incubation with OA and immunostaining for HSP60, mCherry & GFP. **m**, **n**, **o** Representative images (**m**) and automated image analysis of mCherry translocation (**n**) and GFP translocation (**o**) in the cell lines shown in (**g** and **j**), immunostained for HSP60, mCherry and GFP after 3 h incubation with OA in the presence or absence of 1 µM BX795. Data in (**k**, **l**, **n**, **o**) are mean ± s.d. from three independent experiments. *P < 0.05, **P < 0.005, ***P < 0.001, ****P < 0.0001 (two-way ANOVA). ns: not significant. Scale bars: overviews, 10 µm; insets, 2 µm

OPTN and NDP52 recruitment to mitochondria during mitophagy is dependent on ubiquitin binding^14,17,40^. It is therefore unclear how Atg8s can influence OPTN and NDP52 recruitment. We hypothesised that following the initiation of autophagosome biogenesis, the LIR motif within OPTN and NDP52 may enable ubiquitin-independent recruitment of the autophagy receptors via binding to lipidated Atg8s (Fig. 4a; Supplementary Fig. 4a). Thus, we sought to determine whether the LIR motif was sufficient to mediate the translocation of autophagy receptors independently of ubiquitin binding. First, we analysed the localization of OPTN(D474N) and NDP52(C443K) UBD mutants in penta KO cells following PINK1/Parkin mitophagy activation (Fig. 4b). As expected, both mCherry(mCh)-NDP52(C443K) (Fig. 4c) and mCh-OPTN(D474N) (Fig. 4d) failed to translocate to mitochondria after 3 h OA incubation corroborating previous studies^14,41^.

Next, we determined whether UBD mutant autophagy receptors can translocate via their LIR motif during phagophore formation by co-expressing a WT autophagy receptor (Fig. 4e). To prevent the effects of homodimerization between UBD mutant and WT autophagy receptors^42^, cells expressing mCh-OPTN(D474N) were rescued with GFP-NDP52 (Fig. 4g), while cells expressing mCh-NDP52(C433K) were rescued with GFP-OPTN (Fig. 4f). Indeed, the mCh-NDP52(C443K) UBD mutant could translocate to mitochondria when co-expressed with GFP-OPTN (Fig. 4f, k). Likewise, the mCh-OPTN(D474N) UBD mutant translocated to mitochondria in the presence of GFP-NDP52 (Fig. 4g, l). To confirm that the recruitment of UBD mutant autophagy receptors was LIR motif dependent, the experiment was also conducted in cells expressing mCh-OPTN(F178A/D474N) and mCh-NDP52(V136S/C443K) LIR/UBD double mutants (Fig. 4h–j). Consistent with our hypothesis, mCh-NDP52(V136S/C443K) failed to translocate in the presence of GFP-OPTN (Fig. 4i), and mCh-OPTN(F178A/D474N) translocation was significantly defective after 1 h and 2 h OA treatment in the presence of GFP-NDP52 (Fig. 4l). However, after 3 h of OA treatment, mCh-OPTN(F178A/D474N) translocation was observed (Fig. 4j, l). Equivalent translocation of mCh-OPTN(F178A/D474N) did not occur in the absence of GFP-NDP52 (Supplementary Fig. 3b).

NDP52 has been shown to recruit TANK binding kinase (TBK1)^43^, which is known to bind and phosphorylate OPTN^17,24^. We therefore asked whether the recruitment of TBK1 by GFP-NDP52 may account for the residual translocation of mCh-OPTN(F178A/D474N). Indeed, incubation with the TBK1 inhibitor BX795 abolished the residual recruitment of mCh-OPTN(F178A/D474N) (Fig. 4m, n), without influencing the recruitment of GFP-NDP52 (Fig. 4o). Previous work has shown that TBK1-mediated phosphorylation of OPTN’s UBD promotes mitochondrial recruitment^17^, while phosphorylation of the LIR governs Atg8 affinity^40^. The TBK1-dependent recruitment of mCh-OPTN(F178A/D474N) in Fig. 4m–o, indicates that TBK1 plays an additional role in mediating OPTN recruitment beyond LIR and UBD activity. Taken together, OPTN recruitment during mitophagy additionally involves TBK1 activity along with ubiquitin and Atg8 binding. In contrast, NDP52 relies on ubiquitin and Atg8, but not TBK1 for its recruitment. These results explain why NDP52(V136S) has a greater mitophagy defect than OPTN(F178A) (Fig. 2).

**Fig. 5:**
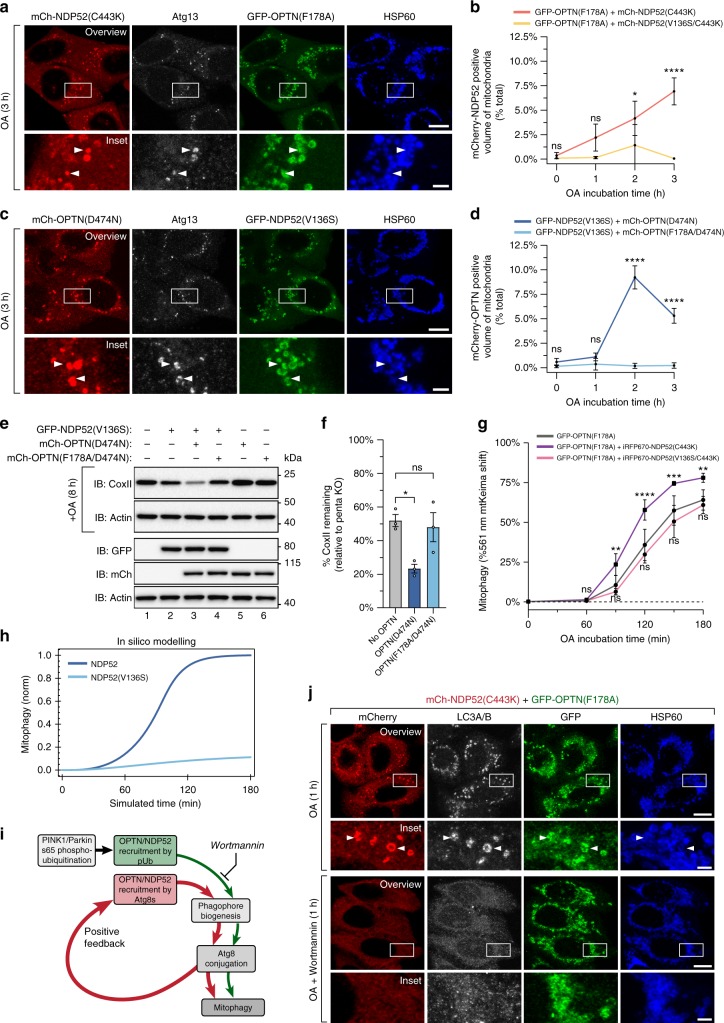
LIR-motif-mediated recruitment of autophagy receptors is required for efficient PINK1/Parkin mitophagy. **a**, **c** Representative images of penta KO cells stably expressing untagged Parkin, with co-expression of either GFP-OPTN(F178A) & mCh-NDP52(C443K) (**a**), or GFP-NDP52(V136S) & mCh-OPTN(D474N) (**c**), immunostained for Atg13, HSP60, mCherry and GFP after 3 h incubation with OA. (Equivalent representative images for mCh-OPTN(F178A/D474N) provided in Supplementary Fig. 3b). **b**, **d** automated image analysis of mCherry translocation in the cell lines shown in (**a**) (**b**) and in (**c**) (**d**) after time-course incubation with OA and immunostaining for HSP60, mCherry and GFP. **e**, **f** Penta KO HeLa cells stably expressing untagged Parkin rescued by stable expression the indicated receptor combinations were analysed by immunoblotting after 8 h incubation with OA (**e**), for quantification of the remaining CoxII levels (**f**). **g** FACS analysis of the percentage of 561 nm mtKeima-positive cells in penta KO HeLa cells stably expressing mtKeima, untagged Parkin and GFP-OPTN(F178A) alone, or with co-expression of either iRFP670-NDP52(C443K) or iRFP670-NDP52(V136S/C443K). (representative FACS plots for mtKeima and iRFP670 expression provided in Supplementary Fig. 1k). **h** In silico modelling of mitophagy using lipidated Atg8 as the surrogate output variable, under wild-type NDP52 or the LIR-mutant NDP52(V136S). (See Methods for details; Supplementary Tables 1–2 for modelled reactions; Supplementary Fig. 4 for overview of the model). **i** The proposed model of LIR-mediated autophagy receptor coalescence in which autophagy receptors are initially recruited by S65 phospho-ubiquitin. The post-initiation lipidation of Atg8s to the expanding phagophore subsequently recruits more autophagy receptors via their LIR motif. The additional autophagy receptors accelerate autophagosome biogenesis to generate a positive feedback loop. **j** Representative images of methanol fixed penta KO cells stably expressing untagged Parkin with co-expression of GFP-OPTN(F178A) & mCh-NDP52(C443K), immunostained for LC3A/B, HSP60, GFP and mCherry after 1 h OA treatment in the presence or absence of 1 µM wortmannin. Data in (**b**, **d**, **f**, **g**) are mean ± s.d. from three independent experiments. *P < 0.05, **P < 0.005, ***P < 0.001, ****P < 0.0001 (two-way ANOVA). ns: not significant. Scale bars: overviews, 10 µm; insets, 2 µm

We next explored the functional significance of LIR-mediated autophagy receptor recruitment by asking if the mitophagy defects of LIR-mutant OPTN(F178A) and NDP52(V136S) (observed in Fig. 2), could be rescued via co-expression of a UBD mutant. First, we tested whether the UBD mutants can be recruited to phagophores when co-expressed with a LIR mutant (Fig. 5). As in Fig. 4, mutant OPTN and NDP52 were cross-combined to prevent the effects of homodimerization^42^. The GFP-OPTN(F178A) LIR mutant was combined with an mCh-NDP52(C443K) UBD mutant (Fig. 5a), and the GFP-NDP52(V136S) LIR mutant was combined with the mCh-OPTN(D474N) UBD mutant (Fig. 5c). Both mCh-NDP52(C443K) (Fig. 5a, b) and mCh-OPTN(D474N) (Fig. 5c, d) were recruited to mitochondria in foci that consistently co-localised with Atg13 labelled phagophores. Recruitment of the UBD mutants to mitochondrial phagophores was dependent on the LIR motif within OPTN and NDP52 (Supplementary Fig. 3c, d). Ubiquitin-independent autophagy receptor translocation via the LIR motif was also observed with p62 (Supplementary Fig. 5c–e), TAX1BP1 (Supplementary Fig. 5f–h), and NBR1 (Supplementary Fig. 5i–k).

Next, we determined whether the recruitment of UBD mutant autophagy receptors was sufficient to rescue the mitophagy defects identified for the LIR mutants OPTN(F178A) and NDP52(V136S) (Fig. 2). The mitophagy defect of GFP-NDP52(V136S) was significantly rescued by co-expression with mCh-OPTN(D474N) but not mCh-OPTN(F178A/D474N), whereas expression of the mCh-OPTN UBD mutants alone did not contribute to CoxII degradation (Fig. 5e, f). In addition, the GFP-NDP52(V136S) mitophagy defect was not rescued by co-expression of mCh-p62(ΔUBD), mCh-TAX1BP1(ΔUBD) or mCh-NBR1(ΔUBD) (Supplementary Fig. 5m, n). The mitophagy efficiency defect of OPTN(F178A) was detected using the mtKeima assay (Fig. 2c–e), therefore this assay was also used for rescue experiments (Supplementary Fig. 1k). The reduced mitophagy efficiency observed for GFP-OPTN(F178A) was significantly rescued by co-expression of iRFP670-NDP52(C443K), but not by the iRFP670-NDP52(V136S/C443K) LIR/UBD double mutant (Fig. 5g). These results demonstrate that LIR-mediated recruitment of OPTN and NDP52 following phagophore formation is critical for efficient PINK1/Parkin mitophagy.

### A positive feedback model of selective autophagy

Our results point toward a model in which OPTN and NDP52 are initially recruited to mitochondria via their UBD. After their recruitment, OPTN and NDP52 promote the recruitment and activation of the ULK1 complex^14^. Subsequent activation of the phosphoinositide 3-kinase complex by ULK1 stimulates local production of PtdIns(3)P at the phagophore nucleation site^29^, which recruits WIPI2b^34^. Recruitment of the Atg8 conjugation machinery (Atg12-Atg5-Atg16L1 complex) by WIPI2b defines the site of LC3/GABARAP lipidation^33^. The Atg8-positive phagophores then recruit additional OPTN and NDP52 via LIR-mediated interactions, which drives further rounds of ULK1 translocation and Atg8 lipidation. This forms an Atg8-dependent positive feedback loop that amplifies the rate of autophagosome biogenesis. The positive feedback loop is lost in the absence of LIR-mediated interactions between autophagy receptors and LC3/GABARAPs (Fig. 5i). This model also provides a mechanistic explanation for the reduced autophagosome size and reduced autophagosome formation efficiency observed during PINK1/Parkin mitophagy in cells lacking Atg8s^37,38^.

To test the positive feedback model, we generated a computational model of PINK1/Parkin activation and autophagosome formation. The in silico model combines the PINK1/Parkin pS65-Ub-dependent positive feedback loop^8,9^, and the LIR-dependent positive feedback loop identified in this study. A mathematical kinetic model of autophagy initiation by NDP52 was formulated using a system of 21 ordinary differential equations (ODEs) with starting conditions and rate constants derived from empirical time-course data (Figs 2, 3, 4). The model scheme is shown in Supplementary Fig. 4 and a detailed model description is presented in Methods. The system of ODEs (Supplementary Tables 1–2) was then numerically solved using Mathematica (Wolfram Research) to quantify total lipidated Atg8 (Atg8PE), which was the surrogate model variable chosen to represent mitophagy output of the system. Upon simulated depolarization of mitochondria (reaction 4), the modelled numerical outputs recapitulated the switch-like response of mitophagy induced by WT NDP52 over time (compare Fig. 5h with 2 h). In contrast, the modelled outputs during mitophagy induction by NDP52(V136S) lacked the positive feedback loop and had significantly diminished mitophagy (Fig. 5h). The PINK1/Parkin mitophagy simulations demonstrated a high degree of robustness, as random perturbation of reaction rate constants had a limited effect on the outcomes (Supplementary Fig. 4d). Taken together, the high level of agreement between in silico model simulations and the biological data strongly support the presence of a positive feedback loop mediated by Atg8s and autophagy receptor LIR binding (Fig. 5i). According to the positive feedback model (Fig. 5i), phosphoinositide 3-kinase (PI3K) inhibitors should block LIR-mediated recruitment of selective autophagy receptors by preventing Atg8 lipidation at initiation sites^28,29,31^. To test the model, the translocation of mCh-NDP52(C443K) (Fig. 5j) and mCh-OPTN(D474N) (Supplementary Fig. 5a) was assessed in the presence of the PI3K inhibitor Wortmannin (Fig. 5j; Supplementary Fig. 5a). Indeed, wortmannin treatment blocked LIR-mediated OPTN and NDP52 recruitment (Fig. 5j; Supplementary Fig. 5a), without affecting upstream ubiquitin-dependent recruitment of LIR-mutant autophagy receptors (Fig. 5i).

In summary, we have discovered a ubiquitin-independent mechanism of autophagy receptor recruitment which amplifies PINK1/Parkin mitophagy signalling by OPTN and NDP52. We argue that cargo selectivity during PINK1/Parkin mitophagy is achieved via de novo autophagosome biogenesis on the surface of mitochondria^14^, but not through LIR-mediated bridging with Atg8s on autophagic membranes. This is supported by reports showing that cells lacking Atg8s successfully sequester mitochondria within autophagosomes^38^, and LC3/GABARAP-lipidation-deficient cells contain autophagic membranes surrounding mitochondria^44^. By clarifying the role of the LIR motif in mitophagy, we have identified an autophagy associated positive feedback loop that drives efficient mitophagy. PINK1/Parkin mitophagy is therefore regulated by two independent but complementary ubiquitin(-like) positive feedback loops. The first is dependent on phospho-ubiquitin generated by PINK1/Parkin^8,9^. The second is dependent on the family of ubiquitin-like Atg8 proteins that are lipidated by the E3 ligase-like Atg12-Atg5-Atg16L1 complex^33,34^. Together, the two positive feedback loops enable OPTN and NDP52 to promote rapid clearance of damaged mitochondria, which would help prevent the release of mitochondrial factors that trigger inflammation and cell death^45,46^.

## Methods

### Cell culture, antibodies and reagents

The penta KO HeLa and hexa KO HeLa cell lines were generated previously^14,38^. All cell lines used in the present study tested negative for mycoplasma contamination. Cell lines were cultured in DMEM supplemented with 10% (v/v) FBS (Cell Sera Australia), 1% penicillin-streptomycin, 25 mM HEPES and GlutaMAX (Life Technologies). Lipofectamine LTX (Life Technologies) transfection reagent was used as per manufacturers specifications. The following rabbit monoclonal and polyclonal antibodies were used: HA (Immunofluorescence (IF), 1:2000; Immunoblotting (IB), 1:1000; #3724, Cell Signaling), FIP200 (IB, 1:1000; #12436, Cell Signaling), LC3A/B (IF, 1:200; #12741, Cell Signaling), Atg13 (IF, 1:200; #13468, Cell Signaling), pS65-Ub (IB, 1:2000; #ABS1513, EMD Millipore), Atg16L1 (IF, 1:200; #8089, Cell Signaling) and Tom20 (IB, 1:4000; #sc11415, Santa Cruz). GFP (IB, 1:1000), MFN1 (IB, 1:5000) and B17.2 L (IF, 1:1000; IB, 1:2000) antibodies were generated previously^47–49^. The following mouse monoclonal antibodies were used: CoxII (IB, 1:1000; #ab110258, Abcam), WIPI2b (IF, 1:500; #ab105459, Abcam), Ub (IB, 1:1000; #MAB1510, EMD Millipore), HSP60 (IF, 1:1000; IB, 1:1000; #128567, Abcam) and Actin (IB, 1:1000; #ab3280, Abcam) and Parkin (IF, 1:200; IB, 1:1000; #sc32282, Santa Cruz). Chicken anti-GFP (IF, 1:500; #a10262, ThermoFisher) and Rat anti-mCherry (IF, 1:500; IB, 1:1000; #M11217, ThermoFisher) were also used.

### Cloning and generation of stable cell lines

Universal vector backbones based on pBMN mEGFP-C1 were generated using the Gibson Cloning kit (New England BioLabs) according to the manufacturer’s instructions: pBMN mCherry-C1, pBMN iRFP670-C1, pBMN HA-C1. The template for production of pBMN mCherry-C1 was pLAMP1-mCherry; a gift from Amy Palmer^50^ (Addgene #45147). The template for production of pBMN iRFP670-C1 was piRFP670-N1; a gift from Vladislav Verkhusha^51^ (Addgene #45457). The HA insert for pBMN HA-C1 was encoded in the cloning primers. Each vector retained sequence homology with the MCS in pBMN mEGFP-C1, to enable Gibson assembly of the same PCR product into all available vectors. This strategy was used to generate GFP-tagged variants of OPTN(F178A), NDP52(V136S), and p62; mCherry tagged variants of OPTN(D474N), OPTN(F178A/D474N), NDP52(C443K), NDP52(V136S/C443K), mCh-p62(ΔUBD), mCh-p62(W340A/ΔUBD), mCh-TAX1BP1(ΔUBD), mCh-TAX1BP1(V142S/ΔUBD), mCh-NBR1(ΔUBD), mCh-NBR1(F562A/Y731A/ΔUBD); HA-tagged variants of LC3A, LC3B, LC3C, GBRP, GBRPL1 and GBRPL2; and iRFP670 tagged variants of OPTN(D474N), OPTN(F178A/D474N), NDP52(C443K), NDP52(V136S/C443K). The templates for OPTN(F178A), OPTN(D474N) and NDP52(V136S) were pHAGE-CMV mCherry-OPTN(F178A), pBMN YFP-OPTN(D474N) and pHAGE-CMV HA-FLAG-NDP52(V136S), respectively, and templates for all other mutants were pBMN mEGFP-OPTN, mEGFP-NDP52, mEGFP-p62, mEGFP-TAX1BP1 and mEGFP-NBR1; all generous gifts from Richard Youle^14^. Point mutants were generated using overlap-extension PCR, and truncation mutants were generated using Gibson assembly. p62(ΔUBD) was generated by truncating p62 at residue 385;^52^ TAX1BP1(ΔUBD) was generated by truncating TAX1BP1 at residue 755;^53^ NBR1(ΔUBD) was generated by truncating NBR1 at residue 906^54^. The primers used for all mutageneses are provided in Supplementary Table 3. Plasmids generated during the study are available from AddGene. Stably transfected cell lines were generated using retroviral systems as described previously^14^, and protein expression levels were normalized between all cell lines by fluorescence activated cell sorting (FACS). Expression level data for the autophagy receptors used in this study are provided in Fig. 5e; Supplementary Fig. 1j, k; Supplementary Fig. 3e; Supplementary Fig. 6.

### Cell treatment and mitophagy induction

For translocation and mitophagy experiments, cells were either left untreated or treated with 10 μM oligomycin (Calbiochem) and 4 μM Antimycin A (Sigma) in fresh growth medium for the times indicated in figure legends. Samples were additionally treated with 10 μM QVD (ApexBio), or 20 μM QVD if the incubation periods exceeded 3 h.

### Immunoblotting

HeLa cells were cultured in 6-well plates for 24 h prior to incubation with either fresh culture medium, EBSS starvation medium, or fresh medium containing 10 μM oligomycin (Calbiochem), 4 μM antimycin A (Sigma) and 10 μΜ QVD (ApexBio) for the times indicated. For immunoblot analysis of cell lysates (Figs 1a, 5e; Supplementary Fig. 5n), cells were lysed in 1 × LDS sample buffer (Life Technologies) supplemented with 100 mM dithiothreitol (DTT; Sigma) and heated to 99 °C with shaking for 7–10 min. For isolation of crude mitochondrial fractions (Supplementary Fig. 3a, f, i), cells were homogenized in 20 mM HEPES (pH 7.6), 220 mM mannitol, 70 mM sucrose, 1 mM EDTA, and 0.5 mM phenylmethylsulfonyl fluoride. After centrifugation of the cell homogenates at 800× *g* for 10 min (4 °C), mitochondria in the post-nuclear supernatant were pelleted by centrifugation at 10,000×*g* for 20 min (4 °C). The mitochondrial pellets were lysed in 1× LDS sample buffer (Life Technologies) supplemented with 100 mM dithiothreitol (DTT; Sigma) and heated to 99 °C with shaking for 7–10 min. Approximately 25–50 μg of protein per sample was separated on 4–12% Bis-Tris gels (Life Technologies) according to manufacturer’s instructions, electro-transferred to polyvinyl difluoride membranes, then immunoblotted using antibodies as indicated. Uncropped data for immunoblots are provided (Supplementary Fig. 7).

### Co-immunoprecipitation

Cells were lysed in lysis buffer (1% TritonX-100, 50 mM Tris-HCl pH 7.4, 150 mM NaCl) supplemented with cOmplete protease inhibitor cocktail (Roche Applied Science). Lysates were cleared by centrifugation (20,000× *g* for 10 min at 4 °C), and subjected to immunoprecipitation using anti-HA magnetic beads (ThermoFisher). The beads were washed three times with wash buffer (0.1% TritonX-100, 50 mM Tris-HCl pH 7.4, 150 mM NaCl), before elution with 1× LDS sample buffer (Life Technologies) and heating to 99 °C with shaking for 7–10 min followed by immunoblot analysis of input and eluted fractions.

### Live-cell and immunofluorescence microscopy

Live-cell imaging samples were prepared by culturing cells in Poly-D-Lysine coated 35 mm FluoroDishes (FD35; World precision instruments) for 48 h before replacing the culture medium with a phenol-free equivalent. For immunofluorescence, cells were cultured on HistoGrip (ThermoFisher) coated glass coverslips for 48 h before experimental treatment. Samples were fixed with 4% (w/v) paraformaldehyde (PFA) in 0.1 M phosphate buffer (15 min). For immunostaining of endogenous Atg8s (Fig. 5j & Supplementary Fig. 5a), samples were fixed with ice-cold methanol^55^. Fixed samples were rinsed three times with PBS, permeabilized with 0.1% (v/v) Triton X-100 in PBS (10 min), then blocked with 3% (v/v) goat serum in 0.1% (v/v) Tween20/PBS (10 min). The samples were incubated with primary antibodies (as indicated in the figure legends) diluted in 3% (v/v) goat serum in 0.1% (v/v) Tween20/PBS for 2 h at room temperature, rinsed three times with PBS, then incubated at room temperature for 1 h with secondary antibodies conjugated to AlexaFluor-405, AlexaFluor-488, Alexa-Fluor-555 or AlexaFluor-647 (ThermoFisher). The coverslips were rinsed three times with PBS, before mounting on slides using a TRIS buffered DABCO-glycerol mounting medium. All samples were imaged in 3D by optical sectioning using an inverted Leica SP8 confocal laser scanning microscope equipped with an ×63/1.40 objective (Oil immersion, HC PLAPO, CS2; Leica Microsystems). Imaging was conducted with a minimum z-stack range of 2.8 µm and a maximum voxel size of 180 nm laterally (x,y) and 320 nm axially (z). All figure images are displayed as z-stack maximum projections. Images for automated analysis were acquired after blindly adjusting the stage position to image three random fields-of-view per coverslip. The randomization procedure was repeated if the field-of-view contained fewer than 30 cells.

### Automated image analysis

All 3D image data were processed and analysed by automated 3D image segmentation using the 3D ROI Manager (v3.93)^56^ and FeatureJ (v3.0.0)^57^ plugins for FIJI (v1.52 h)^58^. The image analysis workflow was divided into three discrete stages; object detection, object measurement and analysis. Detection and segmentation of mitochondria employed a local thresholding strategy. Each image was pre-processed by a 2D sliding paraboloid background subtraction (50 pixel radius), contrast enhancement (0.2% saturation) and a 2D median filter (1.25 px kernel). Seeds for object based segmentation were retrieved by maximum local filter (x,y,z;8,8,4px) before 3D noise reduction (3D median filter; 2 voxels) and automatic 2D local thresholding using the Bernsen method (4 pixel radius). The 3D ROIs were then extracted using a 3D seed-based watershed (5 pixel radius). Detection and segmentation of Atg13 foci, WIPI2b foci, or Atg16L1 foci employed a global thresholding strategy, to account for the lack of foci in the untreated conditions. Linear histogram normalization between experimental repeats was performed by assembling a montage of maximum intensity projections for all images in a given experiment. The montage was used to calculate new minimum and maximum intensity values, which were, respectively, assigned to a given intensity if the number of pixels with that intensity exceeded 0.01% of all pixels in the montage. The histogram of each individual image was then rescaled between these two values before 3D noise filtering (3D median filter; 1.5 voxels) and extraction of the 3D ROIs using a global threshold (96, arbitrary) via simple 3D thresholding (300 voxel maximum size). Each individual 3D ROI was then applied to the original corresponding image to measure the volume and fluorescence intensity of each segmented object in all available channels. All measurements and ROIs were stored for subsequent analyses using Mathematica (version 11).

The mitochondrial volume positive for GFP-tagged autophagy receptors and HA-tagged Atg8s was quantified by inspecting the intensity mean and standard deviation of each segmented object in the corresponding channel. Segmented objects were deemed to be positive for the protein of interest if the mean intensity of the ROI in the corresponding channel was greater than 5 (arbitrary), and the intensity standard deviation exceeded an experiment specific threshold (arbitrary). The following standard deviation thresholds were used for quantification of GFP-tagged receptors: 10, for GFP-OPTN & GFP-OPTN(F178A) in penta KO, GFP-OPTN in penta KO expressing mCh-NDP52 mutants, and GFP-NDP52 in penta KO expressing mCh-OPTN mutants; 15, for GFP-OPTN(F178A) in penta KO expressing mCh-NDP52 mutants, and GFP-NDP52(V136S) in penta KO expressing mCh-OPTN mutants; 25, for GFP-NDP52 and GFP-NDP52(V136S) in penta KO, and GFP-NDP52 in wild type and hexa KO HeLa; 30, for GFP-OPTN in wild type and hexa KO HeLa. The following standard deviation thresholds were used for quantification of mCherry tagged receptors: 30, for mCh-OPTN(D474N) and mCh-OPTN(F178A/D474N) in penta KO expressing GFP-NDP52 or GFP-NDP52(V136S) and mCh-NDP52(C443K) and mCh-NDP52(V136S/C443K) in penta KO expressing GFP-OPTN or GFP-OPTN(F178A). The following standard deviation thresholds were used for quantification of HA-tagged Atg8 proteins: 30, for HA-LC3B in penta KO cells expressing GFP-OPTN or GFP-OPTN(F178A); 20, for HA-LC3C in penta KO cells expressing GFP-NDP52 or GFP- NDP52(V136S). Mitochondrial ROIs 2 voxels or smaller ( ≤ 0.02072 µm^3^) were excluded from all calculations. Foci volume and number for Atg13, WIPI2b and Atg16L1 were quantified by direct measurement of these parameters from the 3D ROIs. The receptor intensity at Atg13 foci was quantified by direct measurement of mean intensity in the receptor channel. The receptor intensity measurements were normalized to values between 0 and 1, respectively, defined by the lowest and highest intensities detected from all samples and repeats (GFP-OPTN & GFP-OPTN(F178A), 0: ⇔ 4.88 and 1: ⇔ 64.28; GFP-NDP52 and GFP-NDP52(V136S), 0: ⇔ 15.05 & 1: ⇔ 193.21). The number of cells per image was quantified manually.

### Mito–Keima autophagosome-lysosome fusion mitophagy assay

Treated cells were trypsinized then pelleted for resuspension in sorting buffer (1 mM EDTA, 10% FCS in 1x PBS) before analysis using a LSRFortessa cell sorter (BD Biosciences). Measurements of lysosomal mito–Keima were made using dual-excitation ratiometric pH measurements at 488 (pH 7) and 561 (pH 4) nm lasers with 695 nm and 670 nm emission filters, respectively. Additional channels were analysed for samples containing GFP (Ex/Em; 488 nm/530 nm) or iRFP670 (Ex/Em; 628 nm/670 nm) for fluorescence compensation. For each sample, 20,000 events were collected and subsequently gated for GFP/Keima double-positive cells, or GFP/Keima/iRFP670 triple-positive cells where relevant. Data were analysed using FlowJo (version 10). The gate used for mtKeima measurements was defined using the untreated sample from each cell line, by aligning the edge of a triangular gate with the untreated bulk population, then moving the gate to encapsulate 99.8% of the population (Supplementary Fig. 1j, k).

### In silico modelling implementation and assumptions

The PINK1/Parkin/NDP52 mathematical model was formulated using ODEs. The model’s schematic diagram containing all model reactions is given in Supplementary Fig. 4. Model ODEs, rate equations and the reference parameter values used for simulations are given in Supplementary Tables 1 and 2. The model was implemented and numerically simulated using Wolfram Mathematica^59^. The model consists of two modules (Supplementary Fig. 4): a PINK1/Parkin module (Supplementary Fig. 4b) and a NDP52 module (Supplementary Fig. 4c). In response to mitophagy stimulation such as OA, PINK1 is assumed to undergo dimerization and becomes activated. It has been reported that dimerized PINK1 is more stable than PINK1 monomers, thus the degradation rate of PINK1 dimer (reaction 3) is assumed to be smaller than that of the monomer (reaction 2). Activated PINK1 subsequently phosphorylates ubiquitin on Serine 65 (reaction 7), which is dynamically opposed by a reverse dephosphorylation reaction by an unspecified enzyme(s). All phosphorylations and similar conversion reactions were modelled with opposing reactions to enable equilibration of the system, as is common practice in computational modelling of biological systems^60–64^. We assume a positive feedback where Parkin once being recruited by phosphorylated ubiquitin is phosphorylated by PINK1 (reaction 11) and phosphorylated Parkin subsequently promote the availability of ubiquitin for phosphorylation by PINK1 (reaction 5)^1,65,66^.

The receptor NDP52 is assumed to bind phosphorylated ubiquitin through its UBD domain (reaction 12). Ubiquitin-bound NDP52 could activate ULK1, which in turn stimulates the conversion of PtdIns to PtdIns(3)P (reactions 14–17). PtdIns(3)P controls Atg8 lipidation by indirectly promoting recruitment of the Atg12-5-16L complex, and Atg8 is deconjugated by Atg4 (reactions 18–19). Importantly, lipidated Atg8 (Atg8PE) can recruit wild-type receptors independently of ubiquitin (reaction 20), because Atg8 can recruit UBD mutant receptors (Figs 4 and 5) and wild-type NDP52 recruitment is defective in hexa KO (Fig. 3). Once NDP52 has been recruited to the phagophore, it is likely it can also interact with ubiquitin (reaction 21) and no evidence exists to suggest otherwise. Similarly, we assume that ubiquitin-bound NDP52 can also bind Atg8 (reaction 13). Atg8-bound NDP52, like ubiquitin-bound NDP52, can also activate ULK1 and thus further promote the production of Atg8PE, generating a positive feedback mechanism. Numerical output of the model used Atg8PE as the surrogate variable for mitophagy.

The parameter values used as the reference set for model analysis and simulations were guided by typical ranges of physiological values and constrained by biologically plausible values^67,68^. Specifically, the rates of protein–protein interactions are given by mass-action (MA) law, and that of (de)phosphorylation reactions are given by Michaelis–Menten (MM) law^60–62^. The *k*_on_ (association) rates are limited by the rate of collisions, which is limited by the rate of diffusion approximately ranging from 0.1 to 10 nM^−1^s^−1,^^69^. Michaelis–Menten constants (*K*_m_) typically vary over a broad range and to explore a wide parameter space, they range from 1 to 1000 (nM). Catalytic constants (*k*_c_) are set between 0.0001 to 1 (s^−1^) and the maximal velocities (*V*_m_) from 0.001 to 10 nMs^−1^. We further constrained the model parameters by fitting the model to the dynamics of PINK1, phosphorylated ubiquitin (pS65-Ub) and mitophagy obtained in Hela cells (Supplementary Fig. 3a). The constrained parameter values are displayed in Supplementary Table 1. To simulate the effect of the LIR-mutant NDP52(V136S), the association constants for reactions 13 and 20 were assigned to zero.

### Statistical calculations

All statistical comparisons were conducted on data originating from three or more biologically independent experimental replicates (as indicated in figure legends), with similar data variances observed between groups. Comparisons between groups were planned prior to statistical testing, and target effect sizes were not pre-determined. No statistical methods were used to determine sample size. All statistical data were calculated and graphed using GraphPad Prism 7. For multi-variable and time-course data, statistical comparisons between groups were performed by two-way ANOVA with post hoc testing by unprotected Fisher’s LSD Test. For data representing a single time-point and condition, statistical comparisons between groups were performed by one-way ANOVA with post hoc testing by unprotected Fisher’s LSD Test. *P* values exceeding 0.05 were considered non-significant.

## Supplementary information

Supplementary Information

## Data Availability

Plasmids generated during the study are available from AddGene. Additional data that support the findings of this study are available from the corresponding authors upon reasonable request.
