# Supplementary material for: LC3/GABARAPs drive ubiquitin-independent recruitment of Optineurin and NDP52 to amplify mitophagy
Source: Nat Commun. 2019 Jan 24;10:408. doi: 10.1038/s41467-019-08335-6 (PMC6345886; doi:10.1038/s41467-019-08335-6)
Supplement: Supplementary file 1 — Supplementary Information [file 41467_2019_8335_MOESM1_ESM.pdf]

Supplementary Information

LC3/GABARAPs drive ubiquitin-independent recruitment of Optineurin and NDP52 to amplify mitophagy

Padman et al.

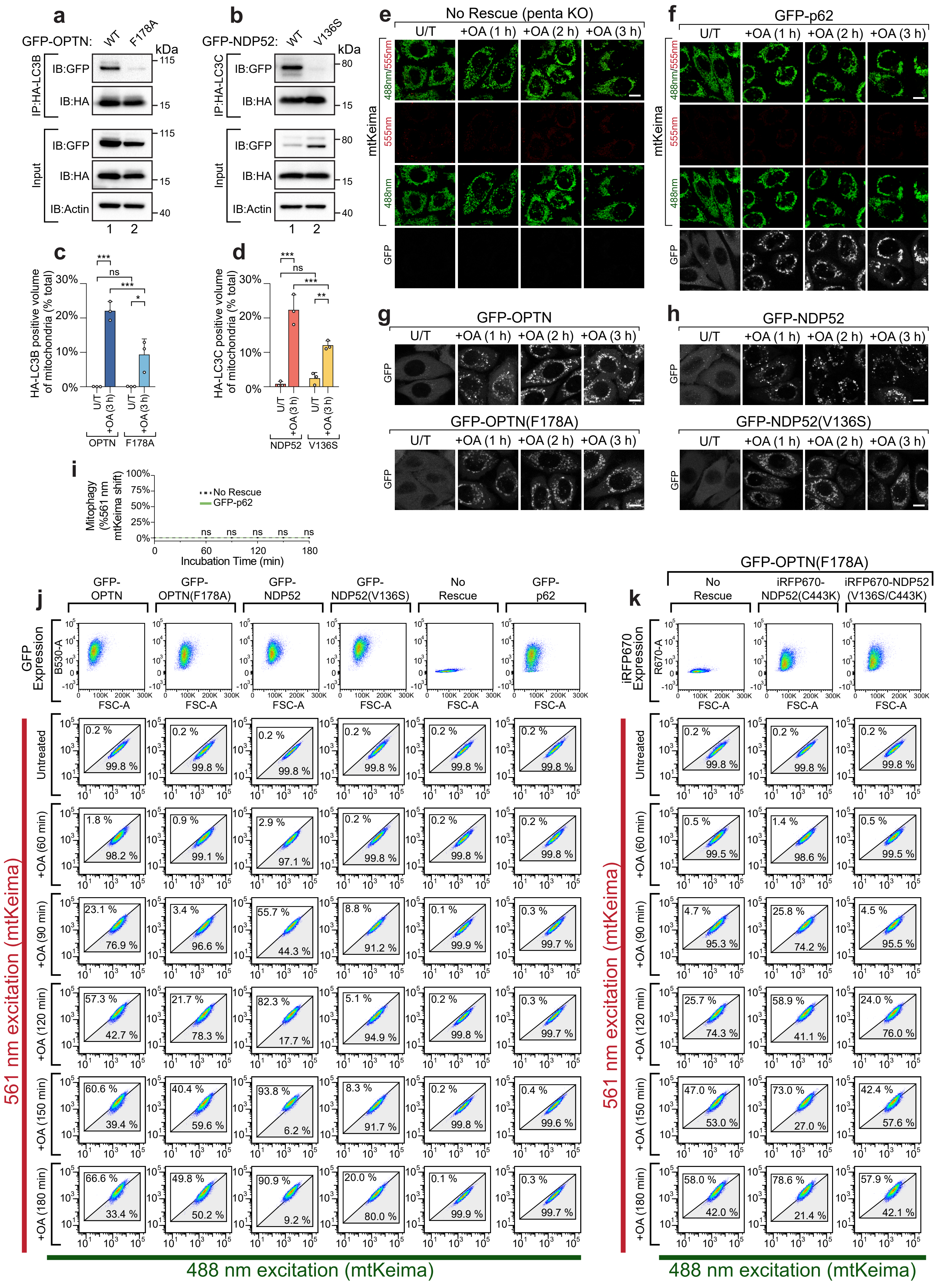

a
GFP-OPTN: WT F178A
IP:HA-LC3B
IB:GFP
IB:HA
Input
IB:Actin
kDa
b
GFP-NDP52: WT V136S
IP:HA-LC3C
c
HA-LC3B positive volume of mitochondria (% total)
OPTN F178A
d
HA-LC3C positive volume of mitochondria (% total)
NDP52 V136S
e
No Rescue (penta KO)
U/T +OA (1 h) +OA (2 h) +OA (3 h)
mtKeima
488nm/555nm
555nm
488nm
GFP
f
GFP-p62
g
GFP-OPTN
GFP-OPTN(F178A)
h
GFP-NDP52
GFP-NDP52(V136S)
i
Mitophagy (%561 nm mtKeima shift)
No Rescue
GFP-p62
Incubation Time (min)
j
GFP-OPTN
GFP-OPTN(F178A)
GFP-NDP52
GFP-NDP52(V136S)
No Rescue
GFP-p62
GFP Expression
B530-A
FSC-A
Untreated
+OA (60 min)
+OA (90 min)
+OA (120 min)
+OA (150 min)
+OA (180 min)
561 nm excitation (mtKeima)
488 nm excitation (mtKeima)
k
GFP-OPTN(F178A)
No Rescue
iRFP670-NDP52(C443K)
iRFP670-NDP52 (V136S/C443K)
iRFP670 Expression
R670-A

Supplementary Figure 1: Quantification of the mitophagy defect in LIR mutant autophagy receptors

a, **b**, Lysates from 1 h OA treated penta KO HeLa cells stably expressing untagged Parkin, with co-expression of HA-LC3B and either WT GFP-OPTN or GFP-OPTN(F178A) (**a**) or HA-LC3C and either WT GFP-NDP52 or GFP-NDP52(V136S) (**b**) were subjected to co-immunoprecipitation using anti-HA beads. Immunoblot analyses of GFP, HA & actin from input lysates are also provided. **c**, **d**, Automated image analysis of penta KO HeLa cells stably expressing untagged Parkin immunostained for HSP60, HA & GFP after incubation with OA (1 h), measuring the mitochondrial translocation of HA-LC3B in the presence of either GFP-OPTN or GFP-OPTN(F178A) (**c**) and mitochondrial translocation of HA-LC3C in the presence of either GFP-NDP52 or GFP-NDP52(V136S) (**d**). **e**, **f**, Representative live-cell images of penta KO HeLa cells stably expressing mtKeima & untagged Parkin without expression of a GFP-tagged autophagy receptor (**e**), or co-expressing GFP-p62 (**f**); imaged after incubation with OA for the indicated times. **g**, **h**, The GFP receptor channels corresponding to Fig. **2c-g**. **i**, FACS analysis of the percentage of 561 nm mtKeima positive cells in penta KO HeLa cells stably expressing mtKeima & untagged Parkin, without expression of a GFP-tagged receptor or co-expressing GFP-p62. **j**, representative GFP & mtKeima FACS plots corresponding to the data shown in Fig. **2e**, **h**, & Supplementary Fig. **1i**. **k**, Representative iRFP670 & mtKeima FACS plots corresponding to the data shown in Fig. **5g**. Data in **c, d, i** are mean ± s.d. from three independent experiments. (two-way ANOVA). ns: not significant. Scale bars: 10 µm. (Uncropped immunoblot data are provided in Supplementary Fig. 7)

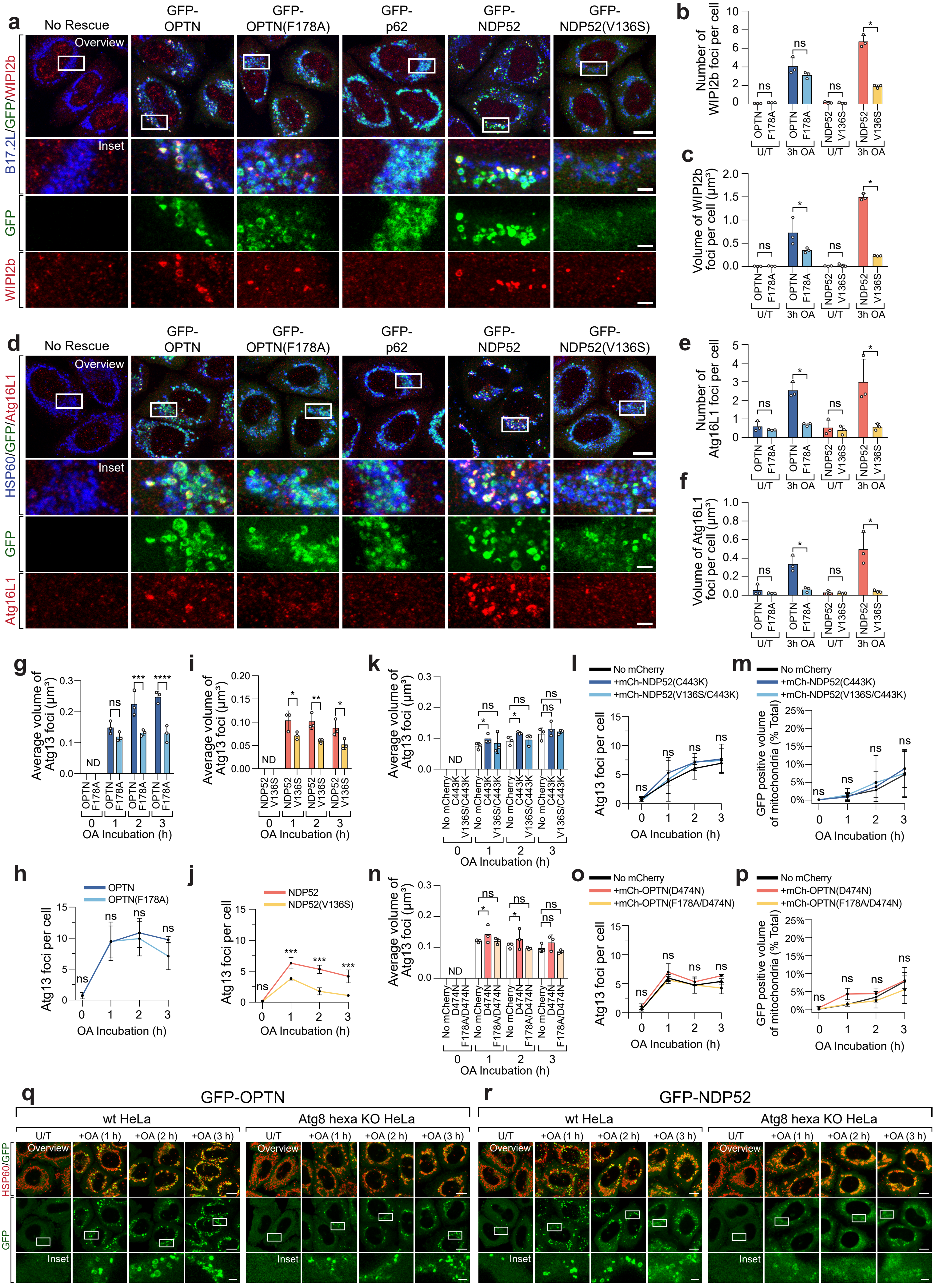

a
No Rescue
GFP-OPTN
GFP-OPTN(F178A)
GFP-p62
GFP-NDP52
GFP-NDP52(V136S)
Overview
Inset
B17.2L/GFP/WIPI2b
GFP
WIPI2b
b
Number of WIPI2b foci per cell
c
Volume of WIPI2b foci per cell (μm³)
d
HSP60/GFP/Atg16L1
Atg16L1
e
Number of Atg16L1 foci per cell
f
Volume of Atg16L1 foci per cell (μm³)
OPTN F178A OPTN F178A NDP52 V136S NDP52 V136S
U/T 3h OA U/T 3h OA
ns
*
g
Average volume of Atg13 foci (μm³)
ND
OA Incubation (h)
i
k
No mCherry C443K V136S/C443K
l
No mCherry
+mCh-NDP52(C443K)
+mCh-NDP52(V136S/C443K)
Atg13 foci per cell
m
GFP positive volume of mitochondria (% Total)
h
OPTN
OPTN(F178A)
j
NDP52
NDP52(V136S)
n
No mCherry D474N F178A/D474N
o
+mCh-OPTN(D474N)
+mCh-OPTN(F178A/D474N)
p
q
GFP-OPTN
r
GFP-NDP52
wt HeLa
Atg8 hexa KO HeLa
U/T
+OA (1 h)
+OA (2 h)
+OA (3 h)
HSP60/GFP

Supplementary Figure 2: Analysis of autophagy initiation and translocation by LIR mutant autophagy receptors
a-c, Representative images (**a**) and automated image analysis of the average number (**b**) and total volume (**c**) of WIPI2b foci per cell after 3 h treatment with OA, in penta KO HeLa cells stably expressing untagged Parkin, rescued by stable expression of the indicated GFP-tagged receptors and immunostained for B17.2L, GFP & WIPI2b. **d-f,** Representative images (**d**) and automated image analysis of the average number (**e**) and total volume (**f**) of Atg16L1 foci per cell after 3 h treatment with OA, in penta KO HeLa cells stably expressing untagged Parkin, rescued by stable expression of the indicated GFP-tagged receptors and immunostained for HSP60, GFP & Atg16L1. **g-j**, Time-course data from the automated image analyses of Atg13 foci in Fig. **3b**, **c**, **e**, **f**. **k**-**p**, Image analyses of the average Atg13 foci size (**k**, **n**), number of Atg13 foci per cell (**l**, **o**), and GFP-tagged autophagy receptor translocation (**m**, **p**) corresponding to the time-course analyses shown in Fig. **5b** (**k**, **l**, **m**) and Fig. **5d** (**n**, **o**, **p**). Quantification of Atg13 foci size in **g**, **i**, **k** & **n** untreated samples were not determined (ND), due to the insufficient number of measurable foci. **q**, **r**, Representative images of wt HeLa & hexa KO HeLa cells stably expressing untagged Parkin and either GFP-OPTN (**q**) or GFP-NDP52 (**r**); immunostained for HSP60 & GFP after time-course incubation with OA. Data in **b**, **c**, **e**, **f**, **g-p** are mean ± s.d. from three independent experiments. *P<0.05, **P<0.005, ***P<0.001, ****P<0.0001 (two-way ANOVA). ns: not significant. Scale bars: overviews, 10 µm; insets, 2µm.

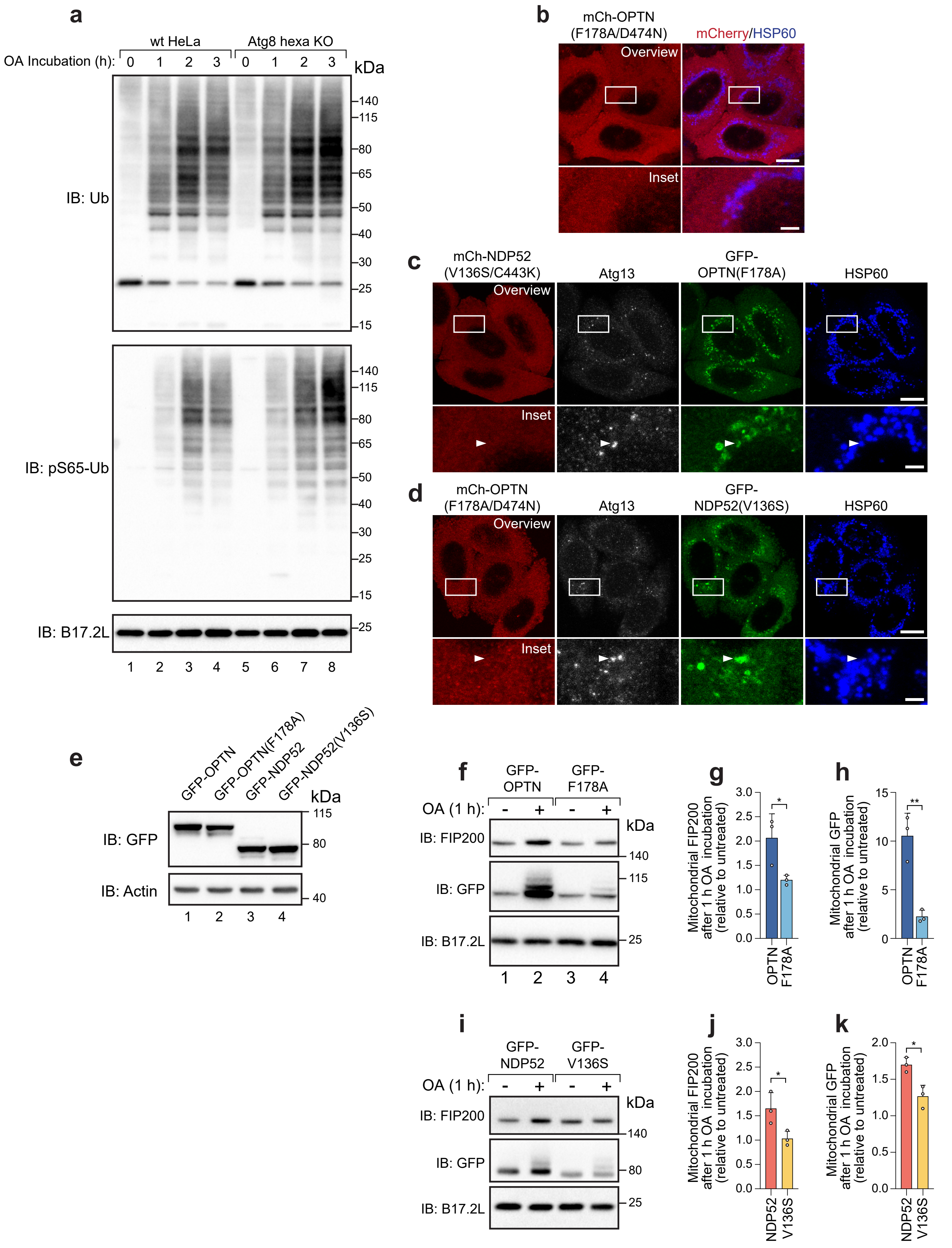

a
wt HeLa
Atg8 hexa KO
OA Incubation (h): 0 1 2 3 0 1 2 3
kDa
IB: Ub
IB: pS65-Ub
IB: B17.2L
140
115
80
65
50
40
30
25
15
1 2 3 4 5 6 7 8
b
mCh-OPTN (F178A/D474N)
mCherry/HSP60
Overview
Inset
c
mCh-NDP52 (V136S/C443K)
Atg13
GFP-OPTN(F178A)
HSP60
Overview
Inset
d
mCh-OPTN (F178A/D474N)
Atg13
GFP-NDP52(V136S)
HSP60
Overview
Inset
e
GFP-OPTN
GFP-OPTN(F178A)
GFP-NDP52
GFP-NDP52(V136S)
kDa
IB: GFP
IB: Actin
115
80
40
1 2 3 4
f
GFP-OPTN
GFP-F178A
OA (1 h): - + - +
IB: FIP200
IB: GFP
IB: B17.2L
kDa
140
115
25
1 2 3 4
g
Mitochondrial FIP200 after 1 h OA incubation (relative to untreated)
OPTN
F178A
*
h
Mitochondrial GFP after 1 h OA incubation (relative to untreated)
OPTN
F178A
**
i
GFP-NDP52
GFP-V136S
OA (1 h): - + - +
IB: FIP200
IB: GFP
IB: B17.2L
kDa
140
80
25
j
Mitochondrial FIP200 after 1 h OA incubation (relative to untreated)
NDP52
V136S
*
k
Mitochondrial GFP after 1 h OA incubation (relative to untreated)
NDP52
V136S
*

Supplementary Figure 3: LIR-mediated translocation of autophagy receptors is ubiquitin-independent

a, Mitochondrial fractions from wildtype and hexa KO HeLa cells after incubation with OA as indicated were immunoblotted for total ubiquitin (Ub), S65 phospho-ubiquitin (pS65-Ub) and B17.2L. **b,** Representative images of penta KO cells stably expressing untagged Parkin, with co-expression of mCh-OPTN(F178A/D474N) (**b**), immunostained for HSP60 & mCherry after 3 h incubation with OA. **c, d,** Representative images of penta KO cells stably expressing untagged Parkin with co-expression of either GFP-OPTN(F178A) & mCh-NDP52(V136S/C443K) (**c**), or GFP-NDP52(V136S) & mCh-OPTN(F178A/D474N) (**d**), immunostained for Atg13, HSP60, mCherry & GFP after 3 h incubation with OA. **e**, immunoblot analysis of GFP-tagged autophagy receptor expression in untreated penta KO HeLa cells stably expressing GFP-OPTN, GFP-OPTN(F178A), GFP-NDP52 or GFP-NDP52(V136S). **f**-**k**, Mitochondrial fractions from Penta KO HeLa cells stably expressing untagged Parkin with co-expression of either GFP-OPTN or GFP-OPTN(F178A) (**f**-**h**) or either GFP-NDP52 or GFP-NDP52(V136S) (**i**-**k**) were isolated after 1 h incubation in the presence or absence of OA. Immunoblot quantitation of FIP200 (**g**, **j**) and GFP-tagged receptors (**h**, **k**) for analysis of enrichment in the mitochondrial fraction. Data in **g, h, j, k** are mean ± s.d. from three independent experiments. *P<0.05, **P<0.005, (Unpaired student's T-test; two-tailed). ns: not significant. Scale bars: overviews, 10 µm; insets, 2µm. (Uncropped immunoblot data are provided in Supplementary Fig. 7)

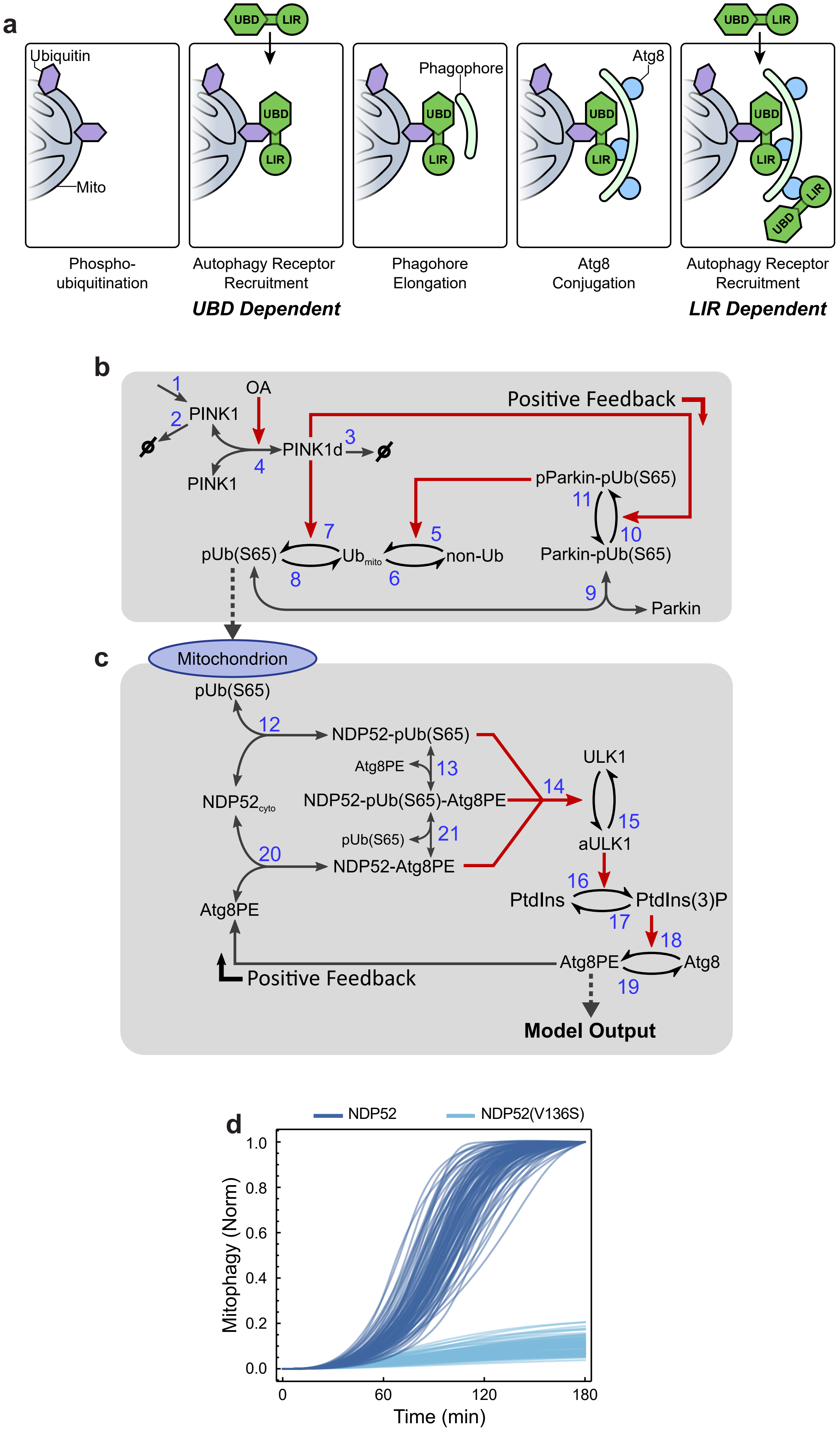
a
UBD
LIR
Ubiquitin
Mito
Phagophore
Atg8
Phospho-ubiquitination
Autophagy Receptor Recruitment
UBD Dependent
Phagohore Elongation
Atg8 Conjugation
Autophagy Receptor Recruitment
LIR Dependent
b
PINK1
OA
Positive Feedback
PINK1d
pParkin-pUb(S65)
pUb(S65)
Ub mito
non-Ub
Parkin-pUb(S65)
Parkin
c
Mitochondrion
pUb(S65)
NDP52-pUb(S65)
Atg8PE
NDP52 cyto
NDP52-pUb(S65)-Atg8PE
ULK1
aULK1
NDP52-Atg8PE
PtdIns
PtdIns(3)P
Atg8PE
Atg8
Positive Feedback
Model Output
d
NDP52
NDP52(V136S)
Mitophagy (Norm)
Time (min)

Supplementary Figure 4: The LIR-mediated amplification model of autophagosome biogenesis during PINK1/Parkin mitophagy
a, Simplified schematic of multimodal receptor recruitment, in which receptors are initially recruited by phospho-ubiquitin on mitochondria via their UBD. Atg8s are conjugated to the expanding membranes after the initiation of autophagosome biogenesis, resulting in a second round of receptor recruitment via LIR mediated interactions. **b**, **c**, Schematic representation depicting the reactions used to model PINK1/Parkin activation (**b**) and NDP52 activation (**c**) during mitophagy. The prefix “p” and “a” denotes phosphorylated and active proteins respectively. The corresponding numbered reactions (Supplementary Tables **1**, **2**) were implemented as outlined in the Methods. **d**, Robustness tests of the mathematical model, in which the reaction constants were randomly perturbed by up to 20% of their original value.

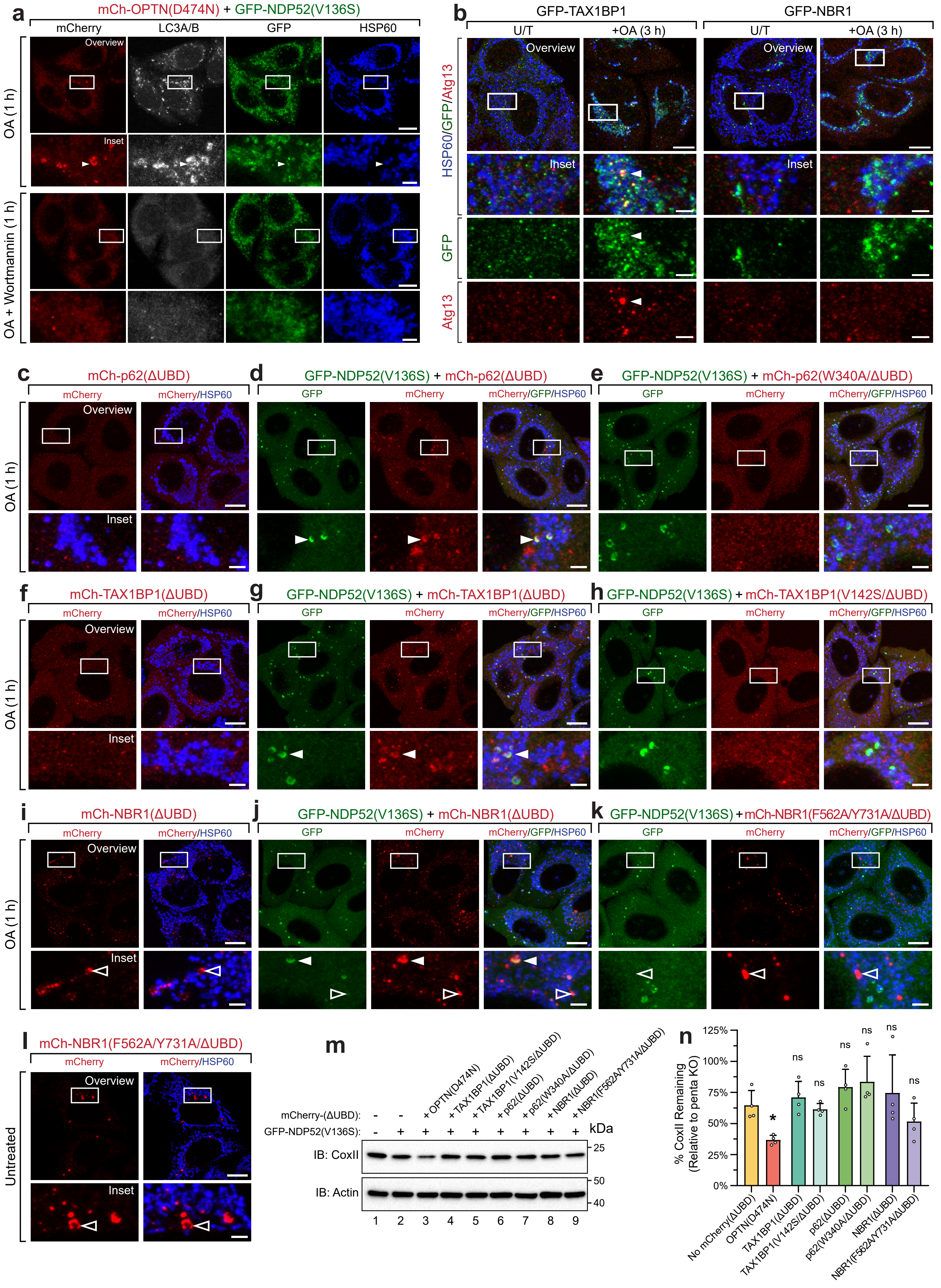
a
mCh-OPTN(D474N) + GFP-NDP52(V136S)
mCherry
LC3A/B
GFP
HSP60
OA (1 h)
Overview
Inset
OA + Wortmannin (1 h)
b
GFP-TAX1BP1
GFP-NBR1
U/T
+OA (3 h)
HSP60/GFP/Atg13
GFP
Atg13
c
mCh-p62(ΔUBD)
mCherry/HSP60
d
GFP-NDP52(V136S) + mCh-p62(ΔUBD)
mCherry/GFP/HSP60
e
GFP-NDP52(V136S) + mCh-p62(W340A/ΔUBD)
f
mCh-TAX1BP1(ΔUBD)
g
GFP-NDP52(V136S) + mCh-TAX1BP1(ΔUBD)
h
GFP-NDP52(V136S) + mCh-TAX1BP1(V142S/ΔUBD)
i
mCh-NBR1(ΔUBD)
j
GFP-NDP52(V136S) + mCh-NBR1(ΔUBD)
k
GFP-NDP52(V136S) + mCh-NBR1(F562A/Y731A/ΔUBD)
l
mCh-NBR1(F562A/Y731A/ΔUBD)
Untreated
m
mCherry-(ΔUBD):
GFP-NDP52(V136S):
+OPTN(D474N)
+TAX1BP1(ΔUBD)
+TAX1BP1(V142S/ΔUBD)
+p62(ΔUBD)
+p62(W340A/ΔUBD)
+NBR1(ΔUBD)
+NBR1(F562A/Y731A/ΔUBD)
kDa
IB: CoxII
IB: Actin
25
50
40
1 2 3 4 5 6 7 8 9
n
% CoxII Remaining (Relative to penta KO)
125%
100%
75%
50%
25%
0%
ns
*
No mCherry(ΔUBD)
OPTN(D474N)
TAX1BP1(ΔUBD)
TAX1BP1(V142S/ΔUBD)
p62(ΔUBD)
p62(W340A/ΔUBD)
NBR1(ΔUBD)
NBR1(F562A/Y731A/ΔUBD)

Supplementary Figure 5: p62, TAX1BP1 & NBR1 are capable of LIR-mediated translocation during PINK1/Parkin mitophagy
a, Representative images of methanol fixed penta KO cells stably expressing untagged Parkin with co-expression of GFP-NDP52(V136S) & mCh-OPTN(D474N), immunostained for LC3A/B, HSP60, GFP & mCherry after 1 h OA treatment in the presence or absence of 1 µM wortmannin. **b**, Representative images of penta KO HeLa cells stably expressing untagged Parkin rescued by expression of the indicated GFP-tagged receptors; immunostained for Atg13, HSP60 & GFP after treatment with OA for 3 h. **c**-**k**, Representative images of penta KO HeLa cells stably expressing untagged Parkin, with co-expression of either mCh-p62(ΔUBD) (**c**), GFP-NDP52(V136S) & mCh-p62(ΔUBD) (**d**), GFP-NDP52(V136S) & mCh-p62(W340A/ΔUBD) (**e**), mCh-TAX1BP1(ΔUBD) (**f**), GFP-NDP52(V136S) & mCh-TAX1BP1(ΔUBD) (**g**), GFP-NDP52(V136S) & mCh-TAX1BP1(V134S/ΔUBD) (**h**), mCh-NBR1(ΔUBD) (**i**), GFP-NDP52(V136S) & mCh-NBR1(ΔUBD) (**j**), or GFP-NDP52(V136S) & mCh-NBR1(F562A/Y731A/ΔUBD) (**k**), after 3 h OA incubation and immunostaining for HSP60, GFP & mCherry. Examples of LIR mediated receptor recruitment are indicated by solid arrowheads; Examples of basal NBR1 localization are indicated by hollow arrowheads. **l**, Representative images of untreated penta KO HeLa cells stably expressing untagged Parkin and mCh-NBR1(F562A/Y731A/ΔUBD), with basal NBR1 structures indicated. **m**, **n**, Penta KO HeLa cells stably expressing untagged Parkin rescued by stable expression the indicated receptor combinations (shown in **d**, **e**, **g**, **h**, **j** & **k**) were analysed by immunoblotting after 8 h incubation with OA (**m**), for quantification of the remaining CoxII levels (**n**). Representative FACS plots demonstrating the expression level of each receptor provided in Supplementary Fig. 6. Data in **n** are mean ± s.d. from three independent experiments. *P<0.05, (one-way ANOVA). ns: not significant. Scale bars: overviews, 10 µm; insets, 2µm. (Uncropped immunoblot data are provided in Supplementary Fig. 7).

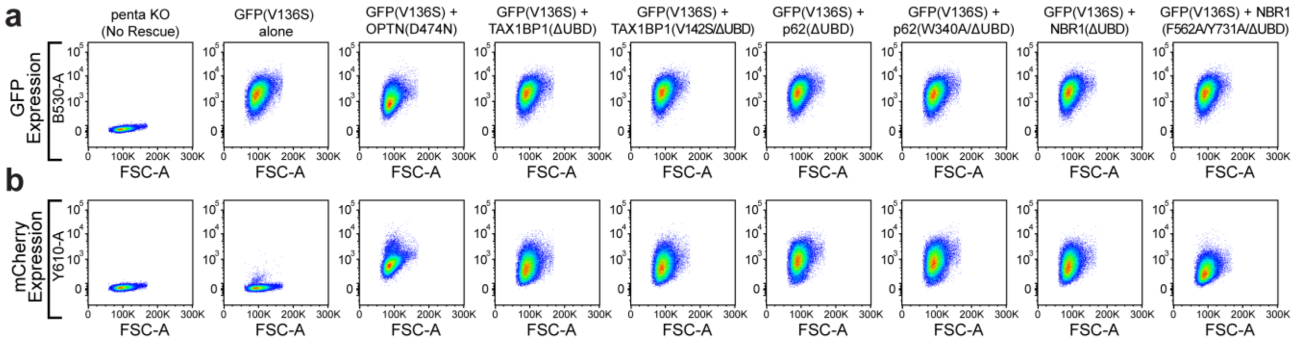

Supplementary Figure 6: Expression levels for mCherry-tagged ΔUBD mutants of TAX1BP1, p62 & NBR1
a, **b**, representative FACS plots for GFP fluorescence (**a**) and mCherry fluorescence (**b**) in penta KO cells or penta KO cells expressing GFP-NDP52(V136S) alone, or expressing GFP-NDP52(V136S) with either mCh-p62(ΔUBD), mCh-p62(W340A/ΔUBD), mCh-TAX1BP1(ΔUBD), mCh-TAX1BP1(V134S/ΔUBD), mCh-NBR1(ΔUBD), or mCh-NBR1(F562A/Y731A/ΔUBD).

Fig. 2a

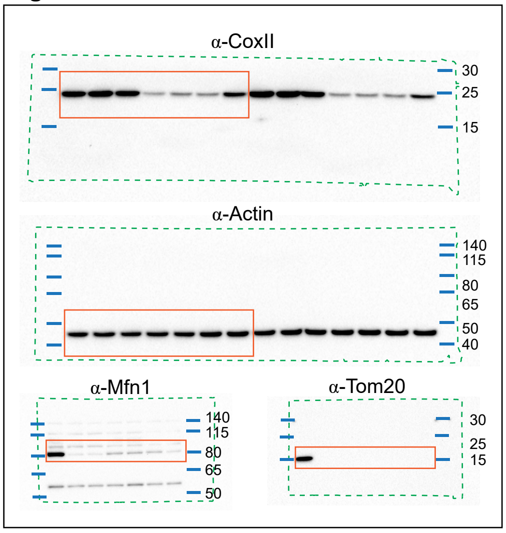

Fig. 5e

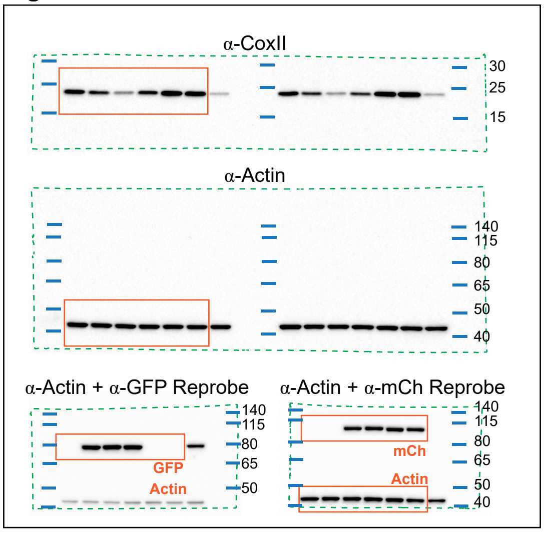

Supplementary Fig. 3a

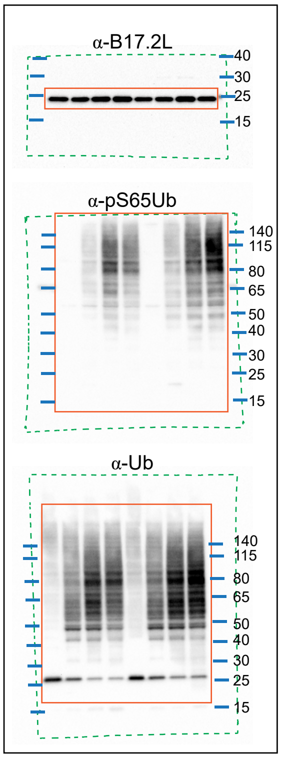

Supplementary Fig. 1a

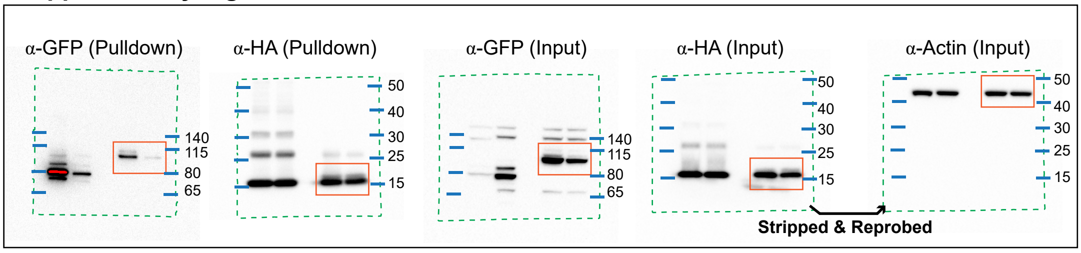

Supplementary Fig. 3e

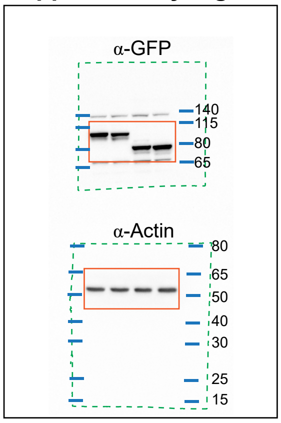

Supplementary Fig. 1b

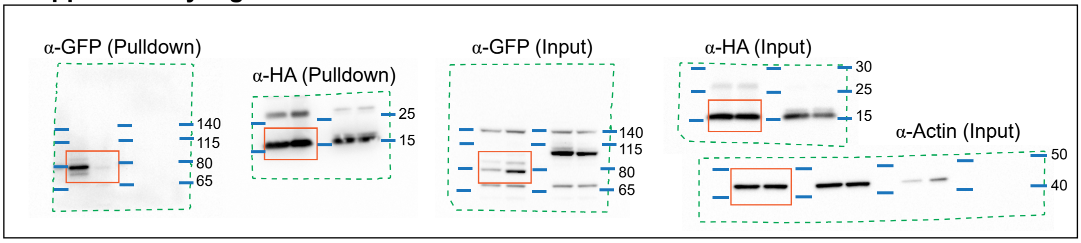

Supplementary Fig. 3f

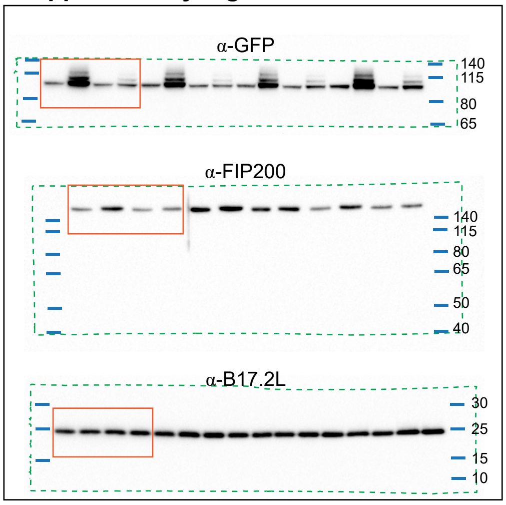

Supplementary Fig. 3i

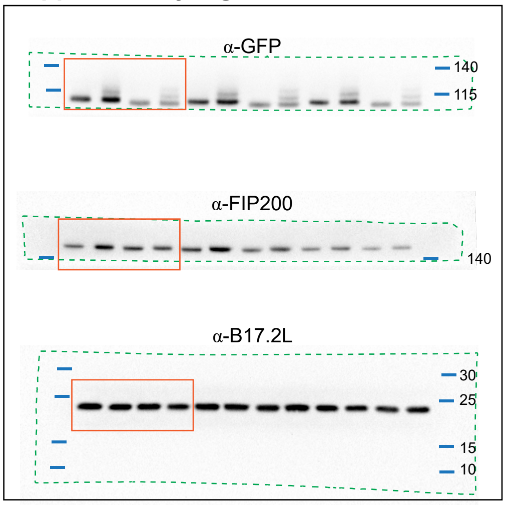

Supplementary Fig. 5m

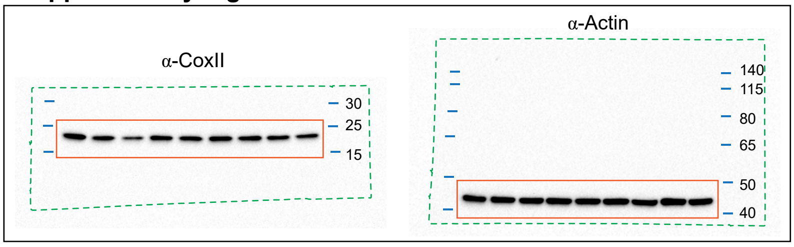

Supplementary Figure 7: Uncropped scans of immunoblots
Molecular weight marker positions indicated by blue lines, cropped regions indicated by orange lines, perimeters of PVDF membranes indicated by dashed green lines.

Supplementary Table 1. Reactions and kinetic rates of the PINK1/Parkin/NDP52 model. Numbered reactions correspond to the model reaction scheme given in Supplementary Fig. 4b, c.

Reaction number	Reactions	Reaction rates	Parameter Values
1	→ PINK1	$v_1 = k_1$	$k_1 = 0.1$
2	PINK1 →	$v_2 = k_2 . [PINK1]$	$k_2 = 0.01$
3	PINK1d →	$v_3 = k_3 . [PINK1d]$	$k_3 = 0.0005$
4	PINK1 + PINK1 ←→ PINK1d	$v_4 = k_{4f} . OA. ([PINK1])^2 - k_{4r} . [PINK1d]$	$k_{4f} = 0.001$; $OA = 10$; $k_{4r} = 0.01$
5	nUb → Ub	$v_{5basal} = \frac{k_{5basal}. [nUb]}{Km_5 + [nUb]}$	$k_{5basal} = 0.1$; $Km_5 = 50$
	nUb → Ub	$v_5 = \frac{k_5. [pParkinpUb]. [nUb]}{Km_5 + [nUb]}$	$k_5 = 0.01$;
6	Ub → nUb	$v_6 = \frac{Vm_6. [Ub]}{Km_6 + [Ub]}$	$Vm_6 = 1$; $Km_6 = 50$
7	Ub → pUb	$v_7 = \frac{k_7. [PINK1d]. [Ub]}{Km_7 + [Ub]}$	$k_7 = 0.01$; $Km_7 = 50$
8	pUb → Ub	$v_8 = \frac{Vm_8. [pUb]}{Km_8 + [pUb]}$	$Vm_8 = 1$; $Km_8 = 50$
9	Parkin + pUb ←→ ParkinpUb	$v_9 = k_{9f} . [Parkin]. [pUb] - k_{9r} . [ParkinpUb]$	$k_{9f} = 0.001$; $k_{9r} = 0.01$
10	ParkinpUb → pParkinpUb	$v_{10} = \frac{k_{10}. [PINK1d]. [ParkinpUb]}{Km_{10} + [ParkinpUb]}$	$k_{10} = 0.008$; $Km_{10} = 50$
11	pParkinpUb→ ParkinpUb	$v_{11} = \frac{V_{11}. [pParkinpUb]}{Km_{11} + [pParkinpUb]}$	$V_{11} = 5$; $Km_{11} = 50$
12	NDP52 + pUb ←→ NDP52pUb	$v_{12} = k_{12f} . [NDP52]. [pUb] - k_{12r} . [NDP52pUb]$	$k_{12f} = 0.01$; $k_{12r} = 0.01$
13	NDP52pUb + Atg8PE ←→ NDP52pUbAtg8PE	$v_{13} = k_{13f}. [NDP52pUb]. [Atg8PE] - k_{13r}. [NDP52pUbAtg8PE]$	$k_{13f} = 0.01$; $k_{13r} = 0.01$
14	ULK1 → aULK1	$v_{14c} = \frac{k_{14}. ([NDP52pUb] + [\mathrm{NDP52Atg8PE}] + [\mathrm{NDP52pUbAtg8PE}]). [ULK1]}{Km_{14} + [ULK1]}$	$k_{14} = 0.0023$; $Km_{14} = 38$
15	aULK1 → ULK1	$v_{15} = \frac{V_{15}. [aULK1]}{Km_{15} + [aULK1]}$	$V_{15} = 0.24$; $Km_{15} = 5$
16	PtdIns → PtdIns(3)P	$v_{16} = \frac{k_{16}. [aULK1]. [PtdIns]}{Km_{16} + [PtdIns]}$	$k_{16} = 0.00018$; $Km_{16} = 2.3$
17	PtdIns(3)P → PtdIns	$v_{17} = \frac{V_{17}. [PtdIns(3)P]}{Km_{17} + [PtdIns(3)P]}$	$V_{17} = 0.211$; $Km_{17} = 85$;
18	Atg8 → Atg8PE	$v_{18} = \frac{k_{18}. [Atg8]. [PtdIns(3)P]}{Km_{18} + [Atg8]}$	$k_{18} = 0.144$; $Km_{18} = 32$
19	Atg8PE → Atg8	$v_{18} = \frac{V_{19}. [Atg8PE]}{Km_{19} + [Atg8PE]}$	$k_{19} = 0.385$; $Km_{19} = 173$
20	NDP52 + Atg8PE ←→ NDP52Atg8PE	$v_{20} = k_{13f}. [NDP52]. [Atg8PE] - k_{13r}. [NDP52Atg8PE]$	Same rates as reaction 13
21	NDP52Atg8PE + pUb ←→ NDP52pUbAtg8PE	$v_{21} = k_{12f}. [\mathrm{NDP52Atg8PE}]. [\mathrm{pUb}] - k_{12r}. [\mathrm{NDP52pUbAtg8PE}]$	Same rates as reaction 12

Supplementary Table 2. The ordinary differential equations and initial conditions of the PINK1/Parkin/NDP52 model. The reaction rates (*v_n*) are given in Supplementary Table 1.

Ordinary Differential Equations	Initial Concentrations (nM)
$\frac{d[PINK1]}{dt} = v_1 - v_2 - v_4$	10
$\frac{d[PINK1d]}{dt} = -v_3 + v_4$	0
$\frac{d[nUb]}{dt} = -v_5 + v_6$	500
$\frac{d[Ub]}{dt} = v_5 - v_6 - v_7 + v_8$	0
$\frac{d[pUb]}{dt} = v_7 - v_8 - v_9 - v_{12}$	0
$\frac{d[Parkin]}{dt} = -v_9$	500
$\frac{d[ParkinpUb]}{dt} = v_9 - v_{10} + v_{11}$	0
$\frac{d[pParkinpUb]}{dt} = v_{10} - v_{11}$	0
$\frac{d[NDP52]}{dt} = -v_{12} - v_{20}$	100
$\frac{d[NDP52pUb]}{dt} = v_{12} - v_{13}$	0
$\frac{d[NDP52pUbAtg8PE]}{dt} = v_{13} + v_{21}$	0
$\frac{d[NDP52Atg8PE]}{dt} = v_{20} - v_{21}$	0
$\frac{d[ULK1]}{dt} = -v_{14} + v_{15}$	100
$\frac{d[aULK1]}{dt} = v_{14} - v_{15}$	0
$\frac{d[PtdIns]}{dt} = v_{17} - v_{16}$	100
$\frac{d[PtdIns(3)P]}{dt} = v_{16} - v_{17}$	0
$\frac{d[Atg8]}{dt} = -v_{18} + v_{19}$	500
$\frac{d[Atg8PE]}{dt} = v_{18} - v_{19} - v_{20}$	0

Table S3. Primers used in this study

Gene	Application	Direction	Sequence
mCherry	Amplification	Fwd	tctagactgccgccaccatgGTGAGCAAGGGCGAGGAG
mCherry	Amplification	Rev	gctcgagatctgagtccggaCTTGTACAGCTCGTCCATGC
iRFP670	Amplification	Fwd	tctagactgccgccaccatgGCGCGTAAGGTCGATCTCACCTC
iRFP670	Amplification	Rev	gctcgagatctgagtccggaGCGTTGGTGGTGGGCGGC
OPTN	Amplification	Fwd	ggactcagatctcgagctcaagctTCCCATCAACCTCTCAGC
OPTN	Amplification	Rev	tcagttatctagatccggtggatccTTAAATGATGCAATCCATCACG
NDP52	Amplification	Fwd	ggactcagatctcgagctcaagcttggGAGGAGACCATCAAAGATC
NDP52	Amplification	Rev	tcagttatctagatccggtggatccTCAGAGAGAGTGGCAGAAC
NDP52(C443K)	Mutagenesis	Fwd	ggactcagatctcgagctcaagcttggGAGGAGACCATCAAAGATCCCCCCAC
NDP52(C443K)	Mutagenesis	Rev	tcagttatctagatccggtggatccTCAGAGAGAGTGTTTGAACACGTGGTC
P62	Amplification	Fwd	actcagatctcgagctcaagcttggGCGTCGCTCACCGTGAAG
P62	Amplification	Rev	agttatctagatccggtggatcctcaCAACGGCGGGGGATGCTT
P62(ΔUBD)	Amplification	Rev	tcagttatctagatccggtggatcctcaGAGATGTGGGTACAAGGCAGC
P62(W340A)	Mutagenesis	Fwd	GAGGAGATGATGACGCGACCCATCTGTCTTC
P62(W340A)	Mutagenesis	Rev	GAAGACAGATGGGTCGCGTCATCATCTCCTC
TAX1BP1	Amplification	Fwd	actcagatctcgagctcaagcttggACATCCTTTCAAGAAGTCC
TAX1BP1	Amplification	Rev	agttatctagatccggtggatcctcaGTCAAAATTTAGAACATTCTGATC
TAX1BP1(ΔUBD)	Amplification	Rev	tcagttatctagatccggtggatcctcaCACCTTCCAGTGACTTTC
TAX1BP1(V142S)	Mutagenesis	Fwd	GAAATTCTGACATGTTAGTGAGCACCACAAAAGCAGGCCTTC
TAX1BP1(V142S)	Mutagenesis	Rev	GAAGGCCTGCTTTTGTGGTGCTCACTAACATGTCAGAATTTC
NBR1	Amplification	Fwd	actcagatctcgagctcaagcttggGAACCACAGGTTACTCTAAATG
NBR1	Amplification	Rev	agttatctagatccggtggatcctcaATAGCGTTGGCTGTACCAG
NBR1(ΔUBD)	Amplification	Rev	tcagttatctagatccggtggatcctcaTGGTGGCCCAGCAAACAG
NBR1(F562A)	Mutagenesis	Fwd	CCCAGGACCTGCTGTCCGCAGAGCTGTTGGATATAAAC
NBR1(F562A)	Mutagenesis	Rev	GTTTATATCCAACAGCTCTGCGGACAGCAGGTCCTGGG
NBR1(Y731A)	Mutagenesis	Fwd	CTTCCTCAGAGGATGCCATCATCATCCTGCC
NBR1(Y731A)	Mutagenesis	Rev	GGCAGGATGATGATGGCATCCTCTGAGGAAG
